# Prevalent knowledge omissions: An unseen risk of LLMs in patient-friendly adaptation of medical literature

**DOI:** 10.1371/journal.pdig.0001739

**Published:** 2026-10-15

**Authors:** Mei Chen, Shibin Wang

**Affiliations:** 1 School of Information Engineering, Minzu University of China, Beijing, China; 2 Key Laboratory of Ethnic Language Intelligent Analysis and Security Governance of MOE, Minzu University of China, Beijing, China; National Tsing Hua University, TAIWAN

## Abstract

Large language models (LLMs) can produce patient-friendly adaptations, such as simplifying specialized medical literature for readers with a middle school education. However, any distortion of knowledge during this process may have serious consequences. Current evaluations overlook such risks. In this study, we proposes a knowledge triplet comparison (KTC) method to assess knowledge distortion by systematically quantifying the retention and alteration of medical knowledge before and after text adaptation. Using the Chemical-Disease Relations dataset, this study evaluates the performance of six LLMs across eight adaptation scenarios (e.g., adjusting reading level, modifying text length) and draws the following key conclusions: First, from a quantitative perspective, knowledge distortion is prevalent in all adaptation scenarios, with the models retaining only approximately half of the original medical knowledge. Additionally, about 10% more knowledge is added. Second, the distribution of knowledge distortion follows a clear categorical distribution pattern, with etiology and pathological mechanisms, as well as complications and risk factors, being the most frequently distortion types. Third, targeted corrections through prompt engineering have no significant impact on the quantity and categorical distribution patterns of knowledge distortion, indicating that knowledge distortion is persistent and regular. This study shows that, in addition to the more obvious risk of hallucinations caused by a small amount of added knowledge, patient-friendly rewrites by LLMs also pose a significant risk of subtle yet potentially serious knowledge omissions. Furthermore, this research reveals the regularity of knowledge distortion, laying the groundwork for further investigation into the patterns of knowledge distortion and the development of solutions to this problem.

## Author summary

Many patients look to medical research articles to better understand their conditions and treatment options. Recently, large language models (LLMs) have been used to rewrite complex medical texts into more accessible language. While this approach may improve understanding, it also raises concerns about whether important medical knowledge is preserved. In this study, we introduce a method called Knowledge Triplet Comparison (KTC) to measure how medical knowledge changes when texts are rewritten for patients. Using data on relationships between chemicals and diseases, we evaluated six widely used LLMs across several common rewriting tasks, such as simplifying language or shortening text. We found that important medical knowledge was frequently altered or lost. On average, only about half of the original knowledge was retained, while some previously unstated knowledge was occasionally introduced. These changes most often affected explanations of disease causes, underlying mechanisms, complications, and risk factors. Notably, even prompts designed to encourage accuracy did not substantially reduce these issues. Our findings highlight a potential risk in using AI to simplify medical knowledge: key details may be silently omitted or changed, which could lead to misunderstanding. Care should therefore be taken when using these tools to communicate medical knowledge to patients.

## 1. Introduction

Patients and their families are increasingly seeking disease-related knowledge, making professional medical literature an essential resource. However, because medical literature is highly specialized, non-expert readers often face significant barriers to understanding. These barriers not only limit patients’ understanding of their conditions but also reduce their ability to engage in treatment decision-making, which ultimately affects healthcare outcomes.

Patient-friendly adaptation refers to rewriting professional medical literature into a more understandable and accessible form based on the patient’s specific circumstances (such as education level), while maintaining the accuracy and integrity of the medical knowledge. In recent years, the rapid development of Large Language Models (LLMs) has created new opportunities for patient-friendly adaptation [1]. Their main advantage is their ability to simplify complex medical literature into patient-friendly language tailored to different audiences, thus making medical knowledge more accessible and improving dissemination efficiency [2]. For example, LLMs can simplify content to a level that is easily understood by individuals with a middle school education, or adjust the length of text as needed [3]. With the increasing prevalence of LLMs, a growing number of patients and their families are independently using LLMs to read and understand specialized medical literature. However, the medical field has an extremely low tolerance for errors in knowledge [4]. Any negligence in the rewritten text could cause patients to form incorrect beliefs, affect their decision-making and even lead to harmful healthcare behaviors [5]. In this study, “knowledge distortion” is defined as an umbrella concept that refers to any change in medical knowledge occurring in the rewritten text relative to the source text. Knowledge distortion can be further divided into two basic types: knowledge omission, which refers to knowledge that is present in the source text but fails to be retained in the rewritten version; and knowledge addition, which refers to the introduction of new knowledge in the rewritten version that lacks support from the source text. In contrast, knowledge consistency refers to knowledge from the source text that is fully preserved during the rewriting process without undergoing substantive modification. Therefore, there is an urgent need for systematic and quantitative analysis to assess the risk of knowledge distortion in LLM-generated patient-friendly medical texts [6].

Adapting medical literature to be patient-friendly can be viewed as a source-conditioned text generation task. Existing research indicates that current LLMs often encounter the issue of ‘hallucination’ in these tasks [7], where they generate content that appears plausible but deviates from the original source material [8]. For instance, in text translation tasks, LLMs may produce translations that are inconsistent with the source semantics due to misunderstandings between the source and target languages [9]; similarly, in summarization tasks, LLMs may generate summaries that contradict key knowledge in the original text [10]. In the medical domain, the accuracy and completeness of medical knowledge are directly linked to patient life and health, making the consequences of knowledge distortion caused by such hallucinations particularly severe [11].

Unfortunately, despite hallucinations being identified in source-conditioned generation, current evaluation methods still fall short of the specific needs of patient-friendly medical adaptation. Existing approaches generally fall into three categories: similarity-based metrics, readability-oriented metrics, and reader-comprehension-based assessments. Similarity-based metrics—such as BLEU [12], ROUGE [13], and BERTScore [14]—rely on n-gram or embedding comparisons [15,16] and can capture surface-level textual resemblance, but they fail to detect logical inconsistencies or fine-grained semantic distortions. Prior work on text simplification has shown that traditional automatic metrics often fail to capture the balance between semantic faithfulness and readability, reflecting superficial similarity rather than true meaning preservation [17]. Readability-oriented metrics, on the other hand, emphasize fluency, coherence, and readability, often assessed by humans or LLMs for grammaticality and consistency [18]; however, they still fall short in evaluating the content quality of generated texts [19,20]. The third category, reader-comprehension-based evaluation, assesses quality based on reader understanding [21,22], yet it likewise cannot specifically reveal knowledge change in simplification.

Overall, while different evaluation approaches capture distinct aspects of text simplification—as demonstrated by comparative studies of scientific text simplification metrics [23]—none directly quantify how medical knowledge changes during LLM-based rewriting. In particular, current approaches cannot systematically measure the retention of key knowledge, identify which categories of medical knowledge (e.g., etiology, symptoms, treatment options) are added, omitted, or altered [24]. These limitations highlight the need for evaluation frameworks that explicitly model medical knowledge structure and fidelity when assessing patient-friendly adaptations of medical literature [7].

To address these shortcomings, this study introduces a novel evaluation method based on knowledge triplet comparison (KTC). Using literature on chemical-disease relationships—critical knowledge often sought by patients—as a case study, we investigate the risks associated with patient-friendly adaptation. Given that the fundamental purpose of patients and families reading such literature is to acquire disease-related knowledge (both direct and indirect), this study employs a knowledge distortion evaluation method focused on the extraction and comparison of disease triplets. This method uses “Disease – Related Relation – Factor” triplets as the fundamental unit of analysis, with a primary focus on the disease as the central element of study. It quantifies knowledge retention and shifts by examining changes in factors related to the disease before and after rewriting. For example, before rewriting, two triplets can be extracted from the original text: [’Disease N’ – ‘Related Relation’ – ‘A’] and [’Disease N’ – ‘Related Relation’ – ‘B’]. After rewriting, two triplets are extracted: [’Disease N’ – ‘Related Relation’ – ‘B’] and [’Disease N’ – ‘Related Relation’ – ‘C’]. This means that after rewriting, the factors related to Disease N lost factor A, added factor C, and retained factor B.

As shown in Fig 1, this evaluation framework consists of three steps: (1) Extraction: Extract triplets from both the original and rewritten medical literature. (2) Comparison: Compare the triplets from the rewritten text with those from the original, identifying added, omitted, and consistent triplets. (3) Classification: Classify the added and omitted triplets based on their medical characteristics, allowing for the identification of potentially misleading content or the absence of critical knowledge. As a novel method introduced in this study, the KTC framework provides a fine-grained, explainable, and quantitative approach for analyzing knowledge transfer fidelity in the adaptation of patient-friendly medical literature. By explicitly modeling knowledge at the triplet level, it enables systematic identification of semantic changes during text rewriting. This study represents an exploratory application of the framework, demonstrating its feasibility and analytical value in assessing knowledge distortion in LLM-generated outputs.

**Fig 1:**
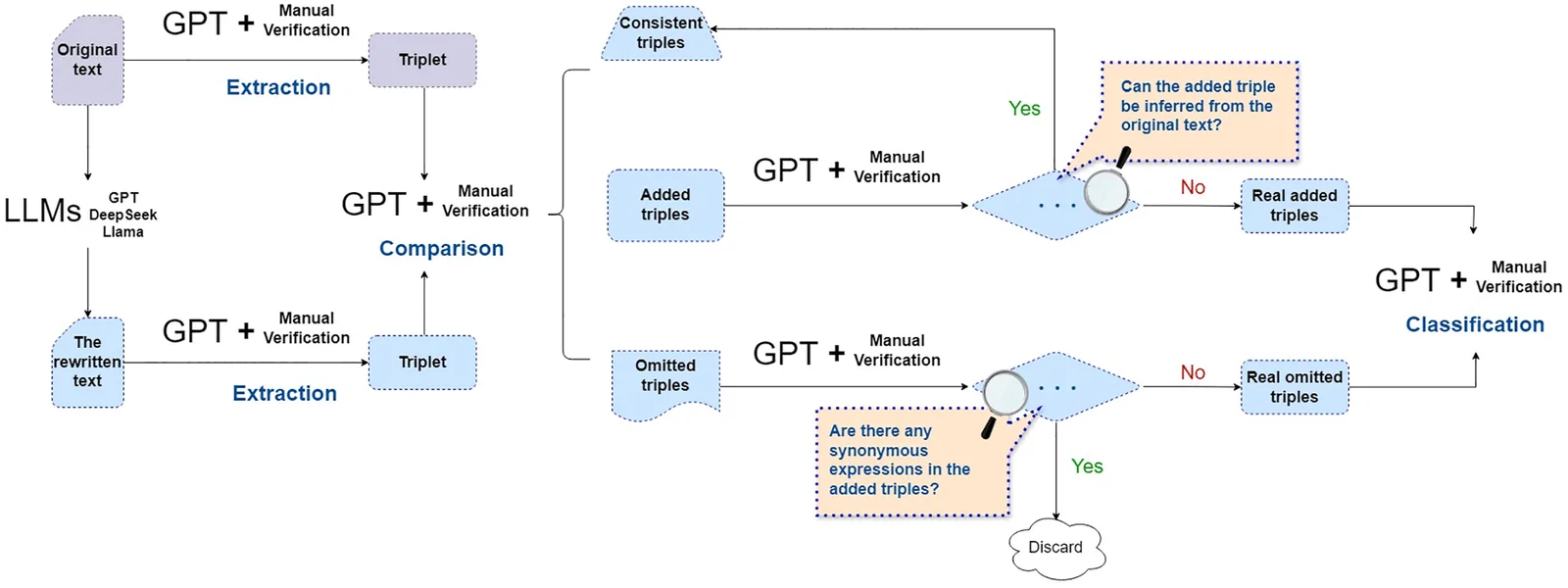
Schematic diagram of knowledge triplet comparison and analysis framework. The model name shown at each stage represents the model used for that specific task. The ‘manual verification’ indicates that the outputs of the corresponding stage were subjected to human validation through random sampling.

This study randomly sampled 100 abstracts from the authoritative CDR dataset [25], ensuring a diverse representation of medical topics. The performance of six mainstream LLMs—ChatGPT-series, Llama-series, and DeepSeek models—was evaluated across eight rewriting scenarios (middle-school reading level, postgraduate school level, expansion, compression, expansion at middle-school level, compression at middle-school level, expansion at postgraduate school level, and compression at postgraduate school level). The research is structured into four phases: (1) Text rewriting: We first generate rewritten versions of the original abstracts using six LLMs across eight predefined scenarios. The quality of the rewrites is then validated to ensure they meet the target readability and length criteria. (2) Knowledge triplet extraction and comparison: Applying the KTC method, we extract “Disease – Related Relation – Factor” triplets from both the original and rewritten texts. By comparing the triplet sets, we obtain the number of changes of added, omitted, and consistent triplets for each rewrite. (3) Categorization analysis of knowledge distortion: we classify the added and omitted triplets respectively based on their medical characteristics to reveal patterns in the types of knowledge most susceptible to distortion. (4) Targeted correction: Based on the distortion categories identified, we design specific prompts to correct the most problematic types of additions or omissions. The corrected texts are then re-analyzed using the KTC framework to assess whether the distortions are mitigated, thereby evaluating the persistence of knowledge distortion and the efficacy of prompt-based correction. In both phases (2) and (3) — knowledge triplet extraction/comparison and categorization analysis — GPT‑4 is used to perform the core tasks. To ensure the reliability of the results, a human validation step is introduced in both phases.

## 2. Results

### 2.1. Text rewriting

The outputs of all rewriting tasks were evaluated to assess whether they met the intended readability and length requirements, with the relevant data provided in S1 Table.

Notably, the evaluation metrics used in this analysis should be interpreted as reference indicators only. For readability, the study employed the Flesch-Kincaid Grade Level (FKGL) as a quantitative indicator. FKGL estimates readability primarily based on sentence length and average word length, but does not adequately capture factors such as domain-specific terminology, required prior knowledge, or reader cognitive load. For example, the CDR dataset used in this study originates from a specialized biomedical research domain and would theoretically correspond to graduate-level readability; however, its FKGL score was 14.6, which is lower than the expected threshold for graduate-level texts. To investigate this further, a manual reading and comparison of the texts before and after rewriting was conducted. This analysis confirmed that the original texts were more specialized and cognitively demanding than the graduate-level rewritten versions. This discrepancy likely reflects limitations of the FKGL metric. Accordingly, FKGL was used in this study primarily as a reference metric for readability adjustment rather than as a comprehensive measure of perceived reading difficulty. Similarly, since LLMs process text in tokens rather than English words, their understanding of text length differs from human conventions; therefore, the length evaluation results are also provided for reference only.

As shown in Fig 2, after rewriting intended to achieve a middle-school readability level, most FKGL scores fell within grades 9–12. This indicates that the rewritten texts generally reached a high-school readability level, which remained higher than the intended middle-school level. For graduate-level rewriting tasks, defined as grades 17–19, approximately half of the rewritten texts achieved FKGL scores within the target range, and most of the remaining texts were close to this range, although a small number still deviated substantially. In terms of length control, all models largely met the preset targets in text-shortening tasks. In expansion tasks, DeepSeek-R1 generated outputs approximately twice the target length, whereas the other models increased the text length but still fell considerably short of the target. Taken together, and in light of the previously noted limitations of the evaluation metrics, these findings support the conclusion that the models have, on the whole, satisfied the core objectives of the patient‑friendly rewriting tasks.

**Fig 2:**
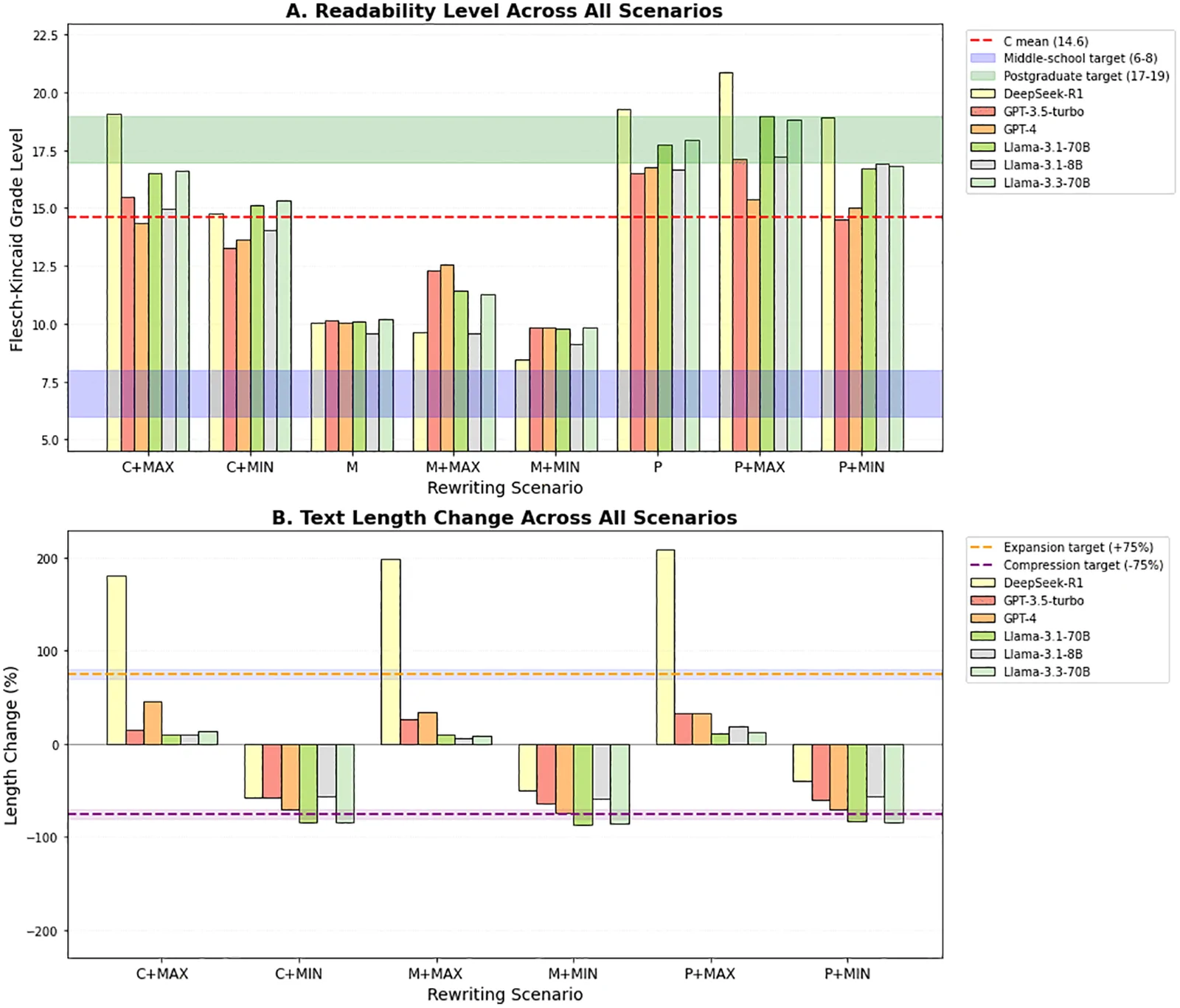
Verification of readability and length across rewriting scenarios. **(A)** Flesch–Kincaid Grade Level (FKGL) of the original and rewritten texts across six LLMs. The blue shaded region denotes the target readability range for middle-school level (Grades 6–8), and the green shaded region denotes the target range for postgraduate level (Grades 17–19). Control conditions(C + MAX, C + MIN) remain close to the original mean readability (14.6). **(B)** Percentage change in text length relative to the original abstracts. The orange dashed line indicates the expansion target (+75%), and the purple dashed line indicates the compression target (−75%). (C: Current Level, M: Middle-School Level, P: Postgraduate Level, MAX: Length Expansion, MIN: Length Reduction).

Overall, in real-world settings, patients are likely to provide broad, directional requests rather than precise specifications. The findings of this study suggest that current models can rewrite texts in accordance with such general patient-oriented requests and show preliminary ability to adjust texts according to broad or underspecified requirements. These results provide preliminary support for the practical feasibility of patient-friendly text rewriting tasks.

### 2.2. Knowledge triplet extraction and comparison

After confirming the effectiveness of the rewriting process, knowledge triplets were extracted from both the original and the rewritten texts. The extracted triplets were then compared and classified into three categories: 1) consistent triplets– triplets present in both the original and rewritten texts, including those identified as semantically equivalent; 2) omitted triplets– triplets present only in the original text; and 3) added triplets– triplets present only in the rewritten text and not semantically equivalent to any triplet in the original text. The numbers of added, omitted, and consistent triplets in the rewritten texts relative to the original texts were then determined (S1 Fig and S2 Fig). Among the omitted triplets, semantic consistency analysis revealed that a very small minority (≤5%) were semantically equivalent to the added triplets; these were excluded from the statistical results after data cleaning. Analysis of the added triplets (S3 Fig) showed that more than half (65% to 86%) essentially represented equivalent expressions of the original text’s semantics. As a result, these were reclassified as consistent triplets. After this correction process, the final counts of truly added, omitted, and consistent triplets were obtained and subsequently analyzed, as shown in Figs 3 and 4.

**Fig 3:**
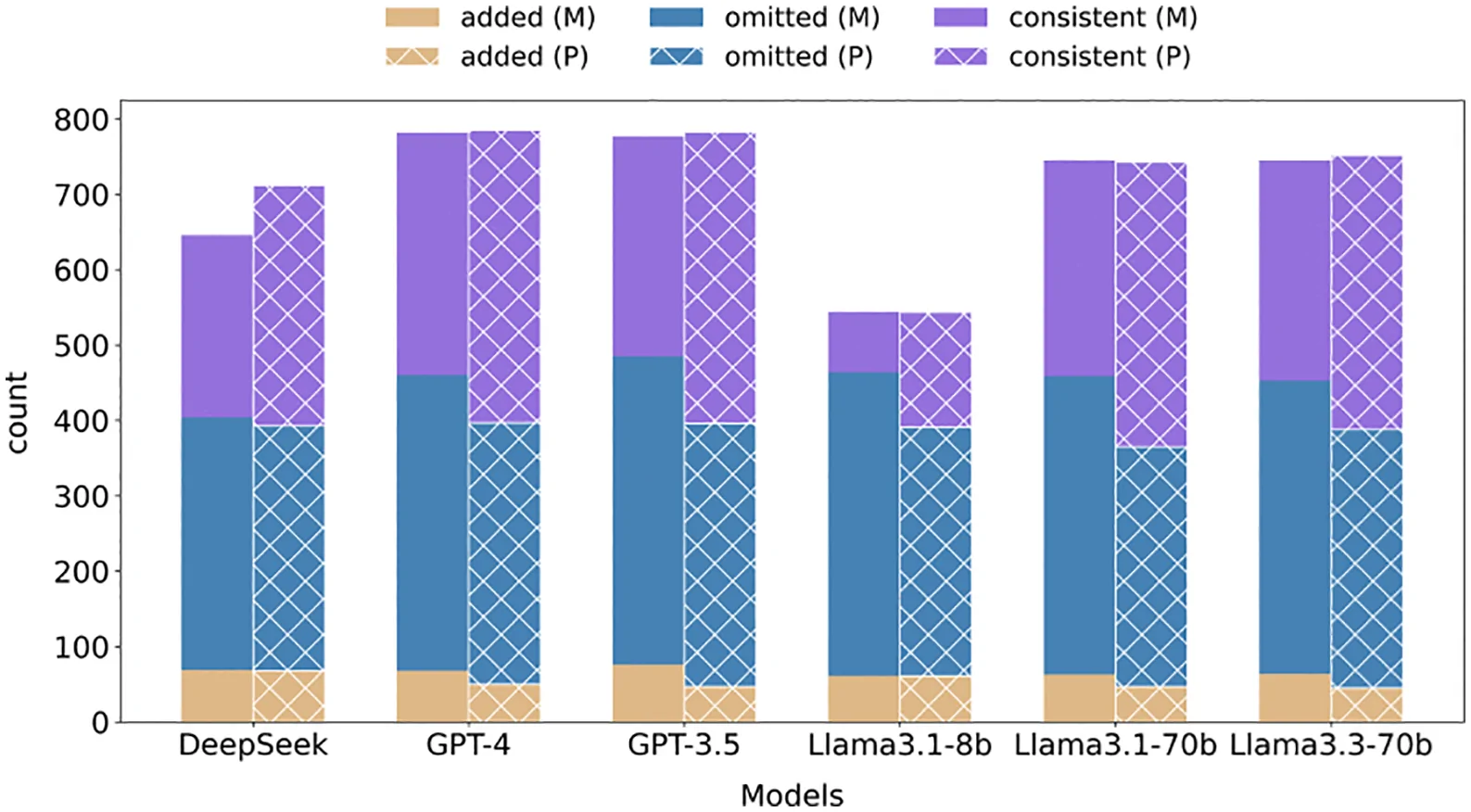
Counts of added, omitted and consistent triplets after readability rewriting. All models exhibit significant loss of original knowledge after rewriting, while the amount of added knowledge they introduce remains uniformly low across all models. (M: Middle-School Level, P: Postgraduate Level).

**Fig 4:**
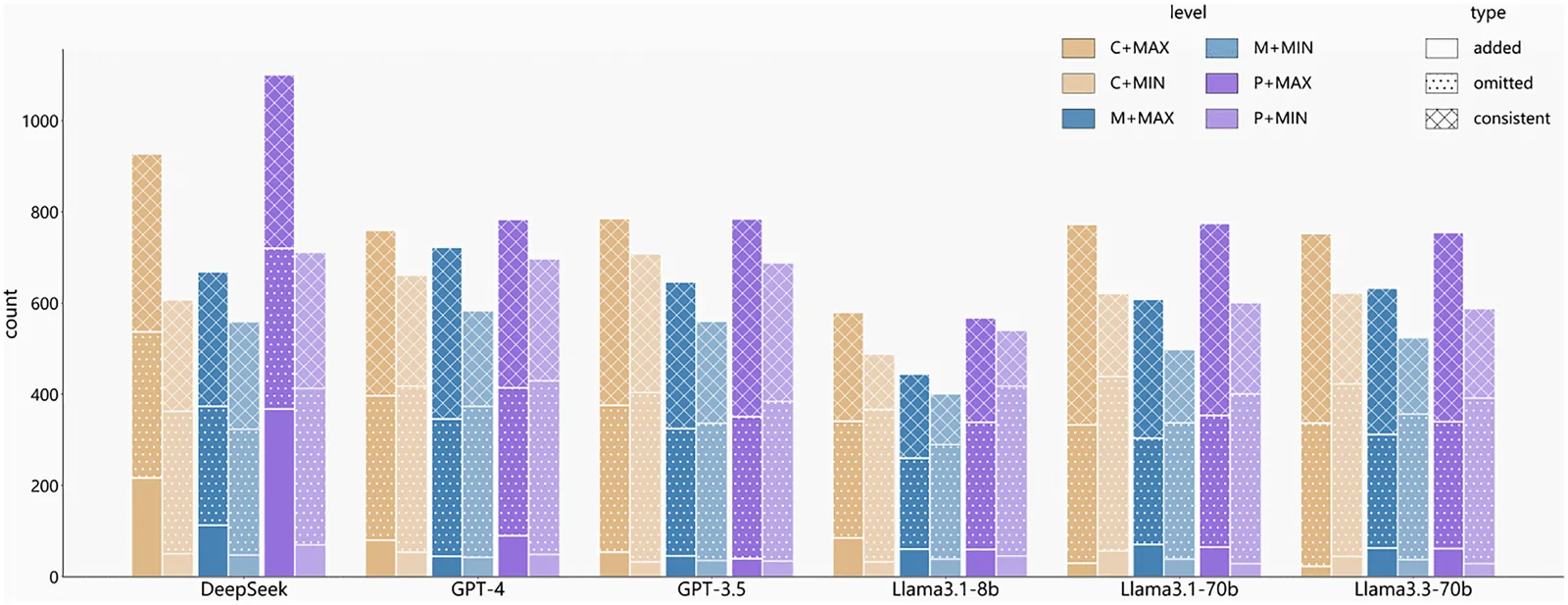
Counts of added, omitted and consistent triplets after text-length rewriting. Across all models, significant original-knowledge loss occurs during both expansion and compression. However, apart from the DeepSeek model, the additional knowledge introduced by other models is minimal. (C: Current Level, M: Middle-School Level, P: Postgraduate Level, MAX: Length Expansion, MIN: Length Reduction).

Fig 3 shows the changes in knowledge triplets after rewriting the original text for two reading levels: middle-school and postgraduate school. The results indicate that: (1) The proportion of original knowledge retained by all models at both reading levels was low (14.71% to 50.87%), with significant knowledge omission observed across all models; (2) Except for Llama3.1-8b, the average knowledge omission (≈47.32%) was roughly equivalent to the preserved content (≈41.62%) for the other models; (3) The average rate of added knowledge across models was generally low (5.98% to 11.21%), with no significant difference between reading levels. Notably, for all models, the amount of knowledge omitted at the middle school reading level was 6.00% to 13.42% higher than at the postgraduate school level. Conversely, the amount of preserved content was lower at the middle school level than at the postgraduate school level. Llama3.1-8b demonstrated significantly lower preservation (14.71% to 28.13%) compared to the other models.

Fig 4 shows the changes in knowledge triplets after rewriting the original text (and versions rewritten for reading level) for length manipulation (expansion and compression). The results indicate that: (1) The proportion of original knowledge retained by models across all scenarios was low (22.59% to 56.92%). All scenarios, including expansion, exhibited a high rate of knowledge omission (31.97% to 68.89%), with omissions often comparable to or exceeding the preserved content; (2) The amount of omitted knowledge in expansion scenarios was slightly lower than in compression scenarios (10.38% to 24.91% lower); (3) Apart from the DeepSeek models, which added substantially more knowledge n during expansion (16.89% to 33.42%), other models added relatively little knowledge across all scenarios (3.05% to 14.66%). Expansion generally resulted in slightly more added knowledge than compression. In higher reading level scenarios, DeepSeek models exhibited significantly more added knowledge.

Overall, the text outputs of various LLMs across different rewriting scenarios exhibit significant semantic deviations from the originals. From a quantitative perspective, knowledge distortion is primarily characterized by a high omission rate (averaging around 50%) and a low addition rate (averaging approximately 10%). Even in expansion tasks, severe knowledge loss persists, indicating systematic knowledge loss during the rewriting process. More critically, the consistency in quantitative trends across various scenarios—such as the consistently high omission rates and limited additions observed under different reading levels and length manipulations—suggests that knowledge distortion in text rewriting is not random noise. Instead, it reflects that current model rewriting is a form of creative restatement rather than faithful transcoding, and that this creative restatement follows certain patterns.

### 2.3. Categorization analysis of knowledge distortion

To analyze patterns of knowledge distortion, the added and omitted triplets were further categorized and statistically analyzed. Through manual reading of a sample of the added and omitted triplets and focusing on factors related to disease, we established a classification for these triplets into five categories: (1) Etiology & Pathophysiology (E class), (2) Symptom (S class), (3) Disease Diagnosis (D class), (4) Therapeutic Drug (T class), and (5) Complication & Risk Factor (C class). As part of this classification, we specified that: If a disease type not mentioned in the original text but appeared in the added triplets, it should be classified into the D class.

Figs 5 and 6 present the classification results for the added and omitted triplets, respectively, while the original data are provided in S2 Table. The results indicate: (1) The categorical distribution for added and omitted triplets show similar patterns. The E and C classes are the most prominent (E ≥ 40%, C ≥ 20%), while the S class consistently remains the lowest (≤8%); (2) Adjustments in reading level and text length did not significantly alter the overall categorical distribution; (3) In expansion scenarios, the counts for most types was higher than in compression; however, the S class increased abnormally in some compression cases, occurring for both added and omitted triplets across multiple models; (4) Two main differences were observed between the categorical distribution for added and omitted triplets: in added triplets, the T and D classes were similar in quantity and relatively high (10.65%: 11.04%), while for omitted triplets, the T and C classes were close (18.21%: 23.71%), yet the D class was significantly lower (5.72%). Additionally, D class occasionally increased in omitted triplets during compression. The result indicates that the categorical distribution of the triplets added and omitted by the models demonstrates discernible consistency and stability—for instance, the high frequency of E and C categories and the consistently low occurrence of the S class. Although certain scenarios exhibit anomalous patterns, this regularity remains broadly observable across various rewriting settings. These findings suggest that knowledge distortion in LLM-based patient-friendly rewriting is not random but is governed by underlying structural biases that follow discernible patterns.

**Fig 5:**
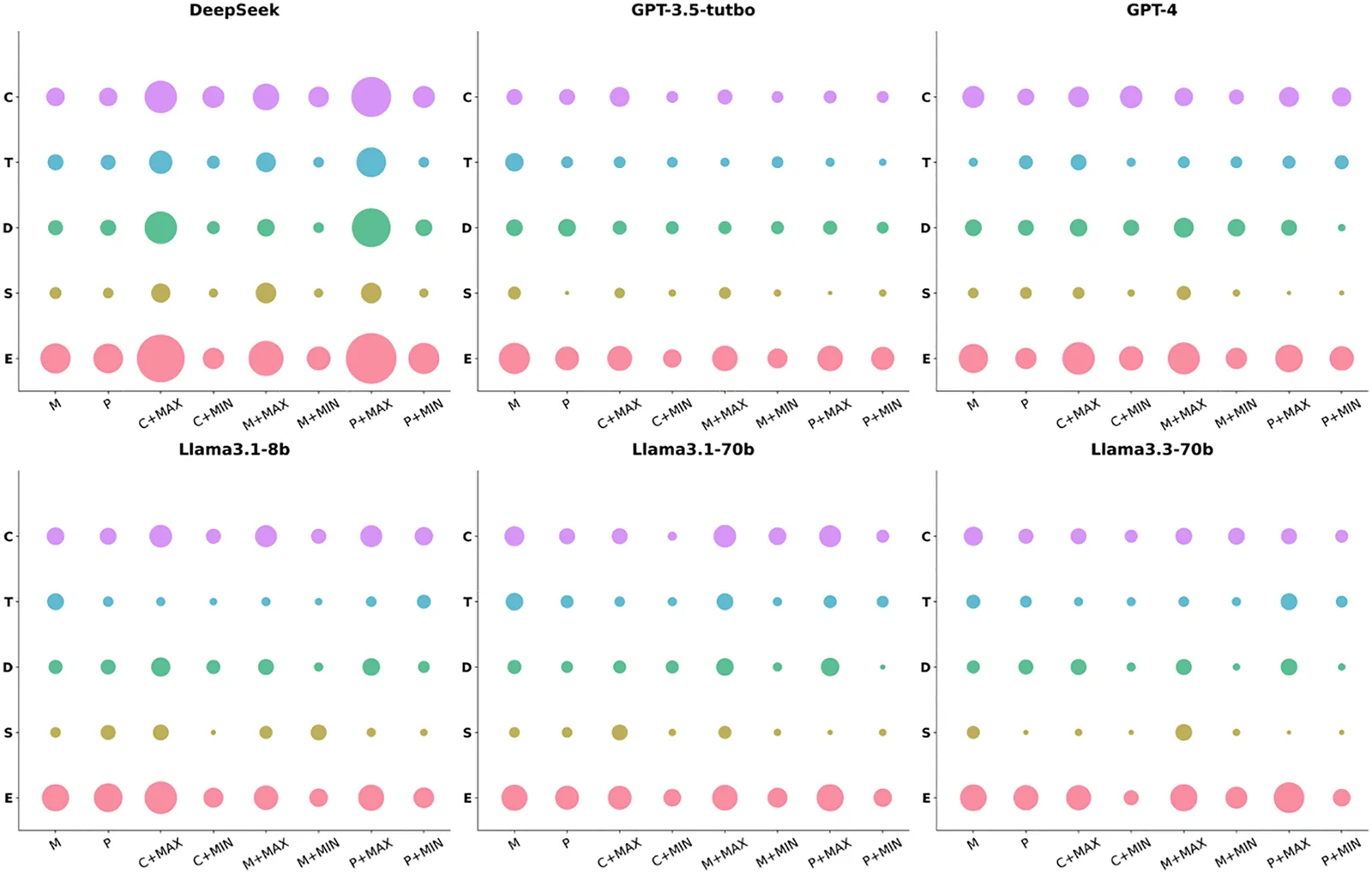
Statistical results of categories for added triplets. The horizontal axis represents text levels (C: current level, M: middle-school level, P: postgraduate level) and text lengths (MAX: expanded, MIN: reduced, C + MAX: expanded at current level, and others following the same pattern). The vertical axis represents types (C, T, D, S, **E)**. Colored bubbles represent different triplet categories, with colors indicating category types and bubble size proportional to the number of occurrences. Larger bubbles therefore represent higher frequencies. In added triplets, categories E and C dominate, with T and D appearing at similar and relatively high frequencies, while S is consistently the smallest category. The overall pattern remains stable across reading level and length adjustments.

**Fig 6:**
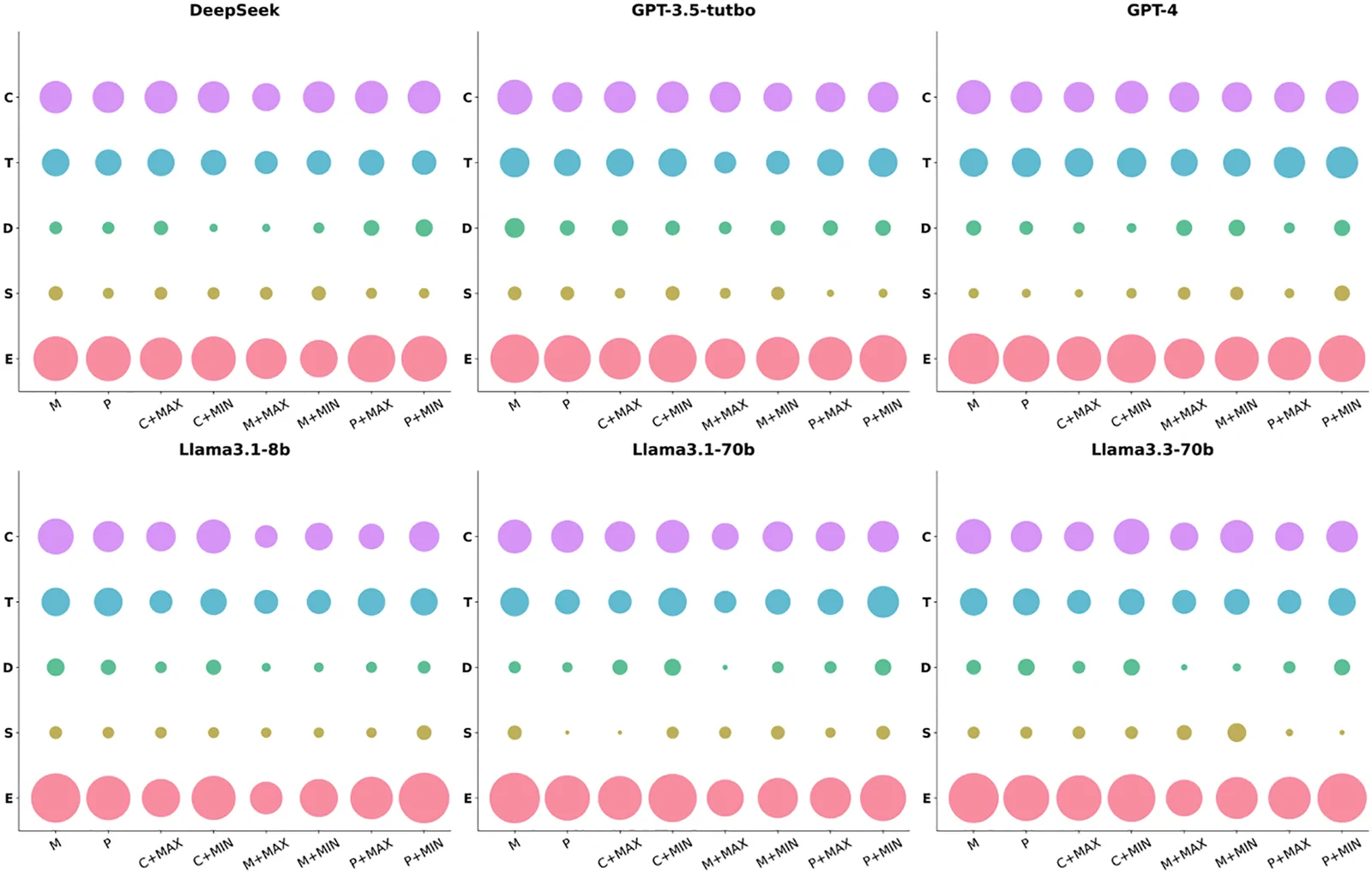
Statistical results of categories for omitted triplets. The horizontal axis represents text levels (C: current level, M: middle-school level, P: postgraduate level) and text lengths (MAX: expanded, MIN: reduced, C + MAX: expanded at current level, and others following the same pattern). The vertical axis represents types (C, T, D, S, **E)**. Colored bubbles represent different triplet categories, with colors indicating category types and bubble size proportional to the number of occurrences. Larger bubbles therefore represent higher frequencies. In omitted triplets, categories E and C also dominate, with T and C close in frequency, but D is notably lower. S remains the smallest category. The distribution is broadly consistent across most rewriting scenarios, reflecting stable structural biases.

### 2.4. Targeted correction

In this section, after incorporating prompts specifically designed for corrective operations based on different distortion categories, the original text was rewritten. The corrected text was then analyzed using the KTC method to evaluate whether targeted correction could mitigate knowledge distortion. Through triplet extraction and semantic consistency analysis of the revised text (S4 Fig), we identified the truly added, omitted, and consistent triplets in the rewritten text (S3 Table).

Fig 7 illustrates the changes in the percentages of added, omitted, and consistent triplets compared to their pre-correction state across different rewriting scenarios for each model. We observed that, except for the Llama3.1-8b model (which showed significant reductions in omissions, all greater than 10%, and corresponding increases in semantic preservation, all greater than 10%, in multiple scenarios), the changes in added, omitted, and consistent triplets for the other models were mostly within ±10%. The overall quantitative trend, where omitted knowledge significantly exceeded added knowledge after rewriting, remained largely unchanged. Figs 8 and 9 display the changes in the proportions of various types for added and omitted triplets post-correction (the specific values in S4 Table, S5 Table, S6 Table and S7 Table). For added triplets, the proportions of Class C and Class E fluctuated noticeably but did not follow a regular pattern; these two types remained dominant. For omitted triplets, the changes in proportions were minor across models and scenarios (most within ±5%), meaning that the overall categorical distribution remained stable.

**Fig 7:**
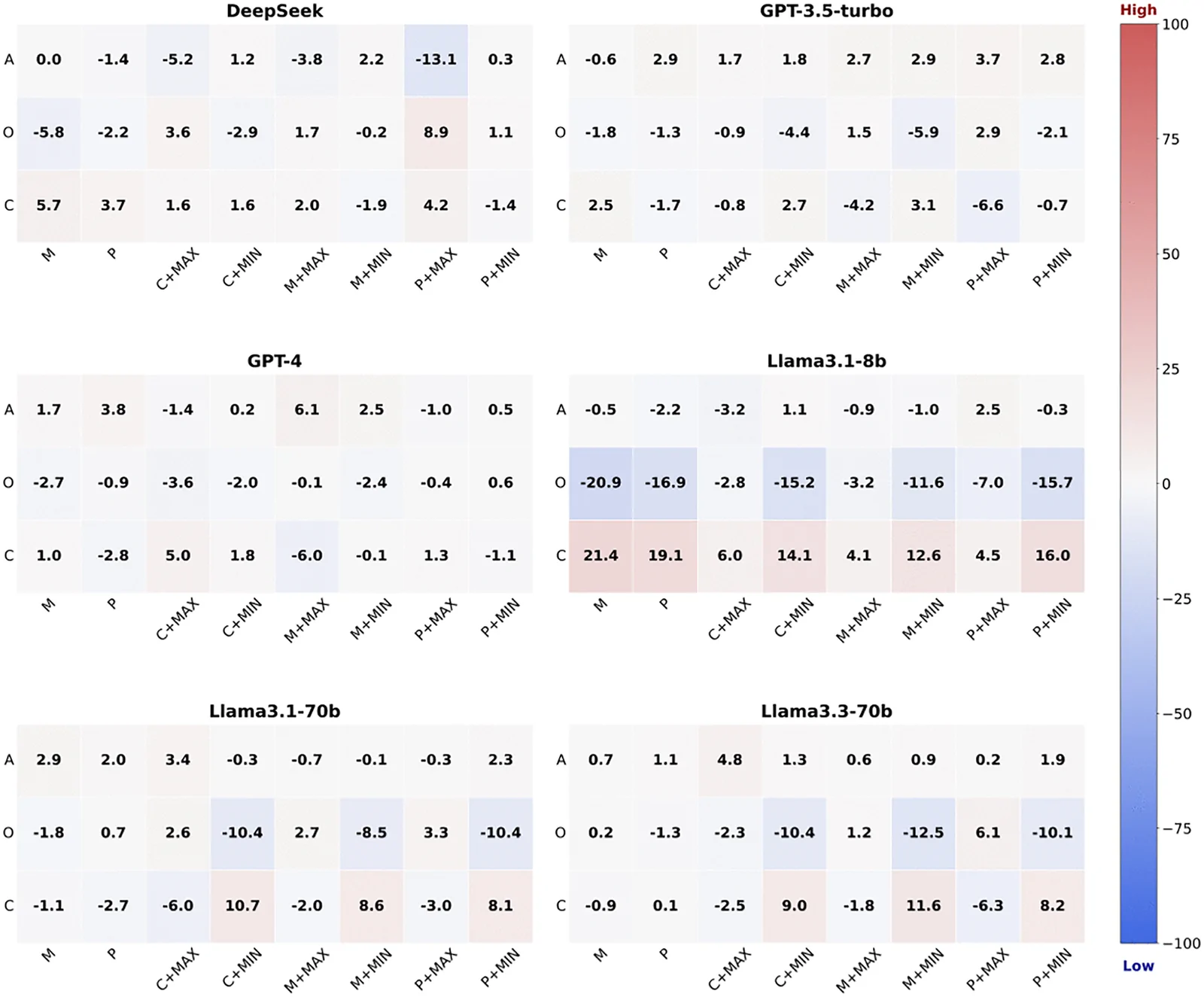
Change (%) in the proportion of added (A), omitted (O), and consistent (C) triplets after correction versus the pre-correction state. The horizontal axis represents text configurations (C: current level, M: middle-school level, P: postgraduate level, MAX: expanded, MIN: reduced, C + MAX: current reading level with expanded text length, and others following the same pattern). The vertical axis labels represent three kinds of triplets: added **(A)**, omitted **(O)**, and consistent **(C)**. The numerical values indicate the difference in the percentage of categories for added triplets after correction compared to the pre-correction baseline. The numerical values indicate the percentage change in each category after correction compared with the pre-correction baseline. Values closer to 0 (lighter colors) indicate smaller changes, while red indicates an increase and blue indicates a decrease. Darker colors represent larger changes in magnitude. Except for Llama3.1‑8b, most models exhibit only small changes after correction. The overall pattern — omission far exceeding addition — remains largely unchanged.

**Fig 8:**
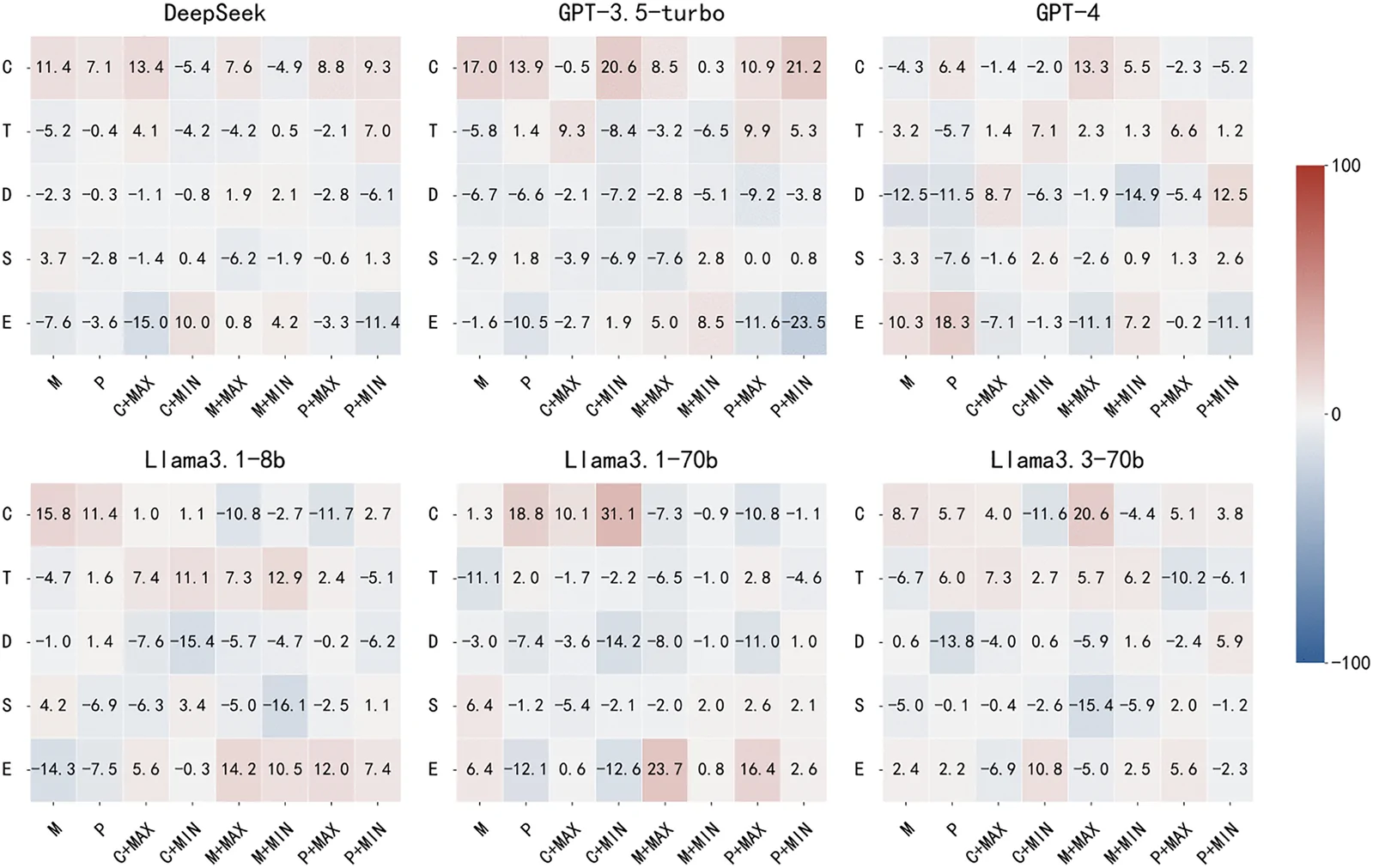
Change (%) in category proportion for added triplets after correction versus the pre-correction state. The horizontal axis represents text configurations (C: current level, M: middle-school level, P: postgraduate level, MAX: expanded, MIN: reduced, C + MAX: current reading level with expanded text length, and others following the same pattern). The vertical axis labels represent five categories: C, T, D, S, **E.** The numerical values indicate the difference i in the category percentages for added triplets after correction compared to the pre-correction baseline. The numerical values indicate the percentage change in each category after correction compared with the pre-correction baseline. Values closer to 0 (lighter colors) indicate smaller changes, while red indicates an increase and blue indicates a decrease. Darker colors represent larger changes in magnitude. Categories C and E continue to predominate among the corrected added triplets, exhibiting conspicuous yet irregular fluctuations, with no stable pattern discernible.

**Fig 9:**
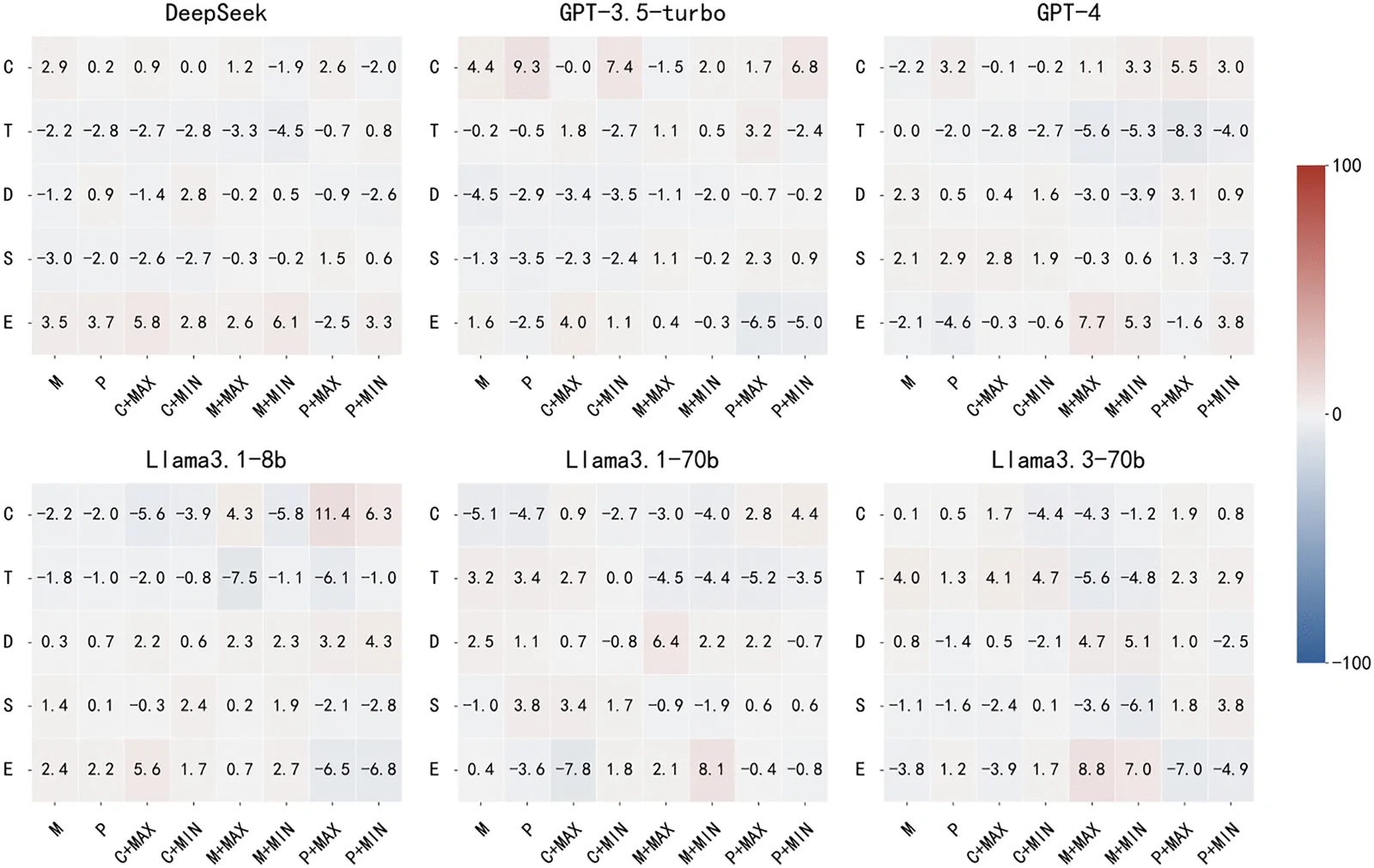
Change (%) in category proportion for omitted triplets after correction versus the pre-correction state. The horizontal axis represents text configurations (C: current level, M: middle-school level, P: postgraduate level, MAX: expanded, MIN: reduced, C + MAX: current reading level with expanded text length, and others following the same pattern). The vertical axis labels represent five categories: C, T, D, S, **E.** The numerical values indicate the difference in the category percentages for omitted triplets after correction compared to the pre-correction baseline. The numerical values indicate the percentage change in each category after correction compared with the pre-correction baseline. Values closer to 0 (lighter colors) indicate smaller changes, while red indicates an increase and blue indicates a decrease. Darker colors represent larger changes in magnitude. Category proportions for corrected omitted triplets show only minor changes, and the overall distribution stays stable.

In conclusion, the targeted corrective operations had little impact on the quantity and categorical distribution patterns of the rewriting results of the LLMs. This indicates that knowledge distortion during the rewriting process by LLMs is highly persistent, suggesting that knowledge distortion is not random but exhibits an inherent regularity.

## 3. Discussions

This study uses CDR texts as a case study and employs a KTC method to conduct a quantitative analysis of knowledge distortion in the patient-friendly adapting of medical literature by LLMs. The study analyzes the severity of knowledge distortion by examining the changes in the number of triplets before and after rewriting. It then investigates the distribution of knowledge distortion types and, finally, explores the persistence of knowledge distortion. The research findings are summarized below: (1) in the rewritten texts across various scenarios, only about half of the knowledge is retained (including expansion scenarios), with approximately 10% of knowledge added. This indicates that patient-friendly adaptation faces a significant risk of knowledge distortion, with the risk of knowledge omission significantly outweighing that of knowledge addition; (2) the categorical distribution patterns of knowledge distortion is not random; rather, it exhibits significant structural patterns. Specifically, both added and omitted triplets primarily relate to Class E and Class C; (3) Adding targeted corrections in the prompts does not change the quantity or categorical distribution patterns of knowledge distortion, indicating that knowledge distortion exhibits persistence and regularity.

Based on these findings, this study suggests that, in the medical domain, current LLM capabilities for patient-friendly adaptation have not yet reached a sufficiently safe and reliable standard for unsupervised use by patients. In addition to the well-known risk of hallucinations, omissions of critical knowledge may pose a more subtle danger, as they could lead patients to overlook important medical facts or risk factors. For example, an LLM-rewritten text might omit clinically relevant relationships described in the original source, thereby altering the completeness or accuracy of the knowledge available to patients. These findings underscore the need to strengthen patient awareness of the limitations of using LLMs to assist in reading medical literature, and to encourage the use of educational materials that have been vetted by authoritative sources. At the same time, the results should not be interpreted as discouraging the use of LLMs in medical communication altogether. A more practical and potentially safer approach is to use LLMs as assistive tools for clinicians, researchers, or medical communicators when preparing patient-friendly materials. In such workflows, professional review remains essential—to verify medical facts, identify omissions, ensure appropriate risk communication, and assess clinical applicability before dissemination. Thus, the primary contribution of this study is to identify potential risks of knowledge omission and semantic distortion in patient-directed LLM adaptations, and to highlight areas that warrant particular attention during expert review.

This study also demonstrates that knowledge distortion exhibits regularity in both its quantity and categorical distribution patterns, along with a persistent nature—that is, targeted corrections fail to alter these inherent patterns. This indicates that patient-friendly rewriting by LLMs constitutes a process of creative restatement that follows certain underlying principles. A key task for future research will be to uncover these principles. To assess the prevalence of such distortion, this study incorporated a diverse set of disease types into the test data, analyzed using a coarse-grained classification framework. Future research aiming to explore the regularity of knowledge distortion in greater depth should focus on more specific sub-domains, developing tailored triplet extraction and classification methods that align with the unique characteristics of sub-domain knowledge to better uncover underlying principles. Notably, based on manual analysis of sample triplets, we observed that in categories where distortion is most prevalent—particularly Class E and Class C—a recurring characteristic is the presence of long-tail knowledge: rare, specialized knowledge that is not widely known. For example, LLMs added the association [“myocardial infarction,” “related to,” “G20210A mutation in the prothrombin gene”]. Since long-tail knowledge often corresponds to novel or niche findings in the literature, its distortion entails particularly severe risks. Given these observations, we argue that future studies should develop fine-grained classification approaches grounded in domain-specific knowledge structures—incorporating explicit markers of long-tail knowledge—to more effectively identify distortion patterns and mitigate associated harms.

This study identifies a significant risk of knowledge distortion when LLMs are used to generate patient-friendly medical texts, with important implications for both public health communication and clinical practice. First, the findings highlight the potential risks of relying directly on LLM-generated patient-friendly texts. Distorted or incomplete knowledge may lead patients to misunderstand their conditions or treatment options, potentially resulting in inappropriate health decisions, delayed treatment, or other harmful outcomes. In extreme cases, such distortions could contribute to clinically serious or even life-threatening consequences. Moreover, the frequency with which these distortions were observed in this study raises concerns about the safety of large-scale deployment of LLM-generated medical communication without adequate safeguards. These results underscore the need for careful oversight, rigorous validation, and well-regulated use of LLMs in healthcare communication. Second, the results may support medical professionals in reviewing LLM-generated patient education materials. The identified patterns of knowledge distortion provide practical cues for detecting and correcting potential inaccuracies, thereby improving the efficiency and reliability of content verification. It should be noted that the clinical examples presented in this study are intended solely to illustrate manifestations of knowledge distortion and do not constitute validated clinical evidence. Further clinical research is required to determine the potential impact of such distortions on patient decision-making and health outcomes.

Although this study provides preliminary evidence of knowledge distortion in patient-friendly text rewriting by LLMs, several limitations should be acknowledged. These limitations primarily pertain to methodological foundations, research design, and the generalizability of the findings.

First, at the methodological level, the conclusions of this study depend to some extent on the performance of GPT-4. The KTC framework is fundamentally based on knowledge triplet comparison, and its reliability therefore relies on the accuracy of triplet extraction, comparison, and classification. In this study, GPT-4 was employed across these stages, selected based on prior literature synthesis and our previous document-level biomedical relation extraction evaluation work [26], which indicated its relatively strong performance. Following the experiments, additional manual sampling verification was conducted, with error rates across different stages estimated to be below 10%. Nevertheless, no automated system can fully eliminate systematic bias. The use of GPT-4 across multiple stages of the pipeline (including both extraction and evaluation) may introduce potential “same-model” bias, whereby shared model characteristics could lead to an underestimation of certain types of errors. This concern is particularly relevant for fine-grained distinctions between added, omitted, and consistent triplets, which rely on subtle semantic judgments. Moreover, triplet extraction constitutes the foundational step of the KTC framework; errors at this stage may propagate through subsequent processes and lead to cascading amplification of inaccuracies. Although both prior studies and our manual evaluation suggest that GPT-4 performs relatively reliably in relation extraction tasks, a degree of uncertainty inevitably remains. In addition, the absence of independent extraction models or formal inter-annotator agreement limits the ability to fully quantify uncertainty in these distinctions. Consequently, the findings of this study should be interpreted as indicative rather than definitive. Future work could enhance the robustness of the KTC framework by incorporating independent extraction models, cross-model validation, or human-verified relation extraction pipelines, thereby reducing model-specific biases and improving the reliability of large-scale analyses of knowledge distortion in medical text simplification.

Second, regarding research design, the sample size and classification system in this study remain exploratory in nature. One hundred medical paper abstracts were selected as the research sample. Preliminary analysis indicated that variability in the observed patterns began to stabilize after approximately 80 documents (S5 Fig). This suggests that the patterns approach relative stability within the scope of this dataset. However, this sample size remains insufficient to comprehensively represent the broader corpus of medical research. Furthermore, the five-category classification system proposed in this study was inductively constructed through preliminary manual review of extracted results combined with medical knowledge, which may introduce a degree of subjectivity. In addition, disease-related triplets newly introduced by the model but absent from the source text are operationally classified under the diagnosis (D) category. We acknowledge that not all such additions necessarily constitute errors, as some may reflect clinically plausible inferences. However, given their small proportion, distinguishing between erroneous fabrication and reasonable inference is beyond the scope of this study and is noted as a limitation for future research. The primary purpose of this classification framework was to explore whether knowledge distortion exhibits structural patterns, rather than to establish a universal theoretical classification system. As sample sizes expand in future research, both the robustness of the observed patterns and the classification framework will require further validation and refinement.

Finally, our findings indicate that introducing targeted corrective prompts yields only limited improvement in the quality of patient-friendly rewriting. This suggests that the quality of such rewriting depends more on the intrinsic mechanisms of the models than on superficial adjustments to prompt phrasing. Of course, this conclusion should be interpreted as a general trend, and the possibility of different outcomes under specific prompt structures or stronger constraints cannot be ruled out. In this study, we employed two types of prompts. The first type—rewrting prompts—was designed to simulate real-world usage scenarios and reflect the actual needs of patients when seeking knowledge. These prompts instructed the model to adjust the output according to a specified reading level or text length. The second type—corrective prompts—was designed based on the typical distortion patterns identified in our preliminary analysis, and required the model to adhere to the relevant knowledge in the source text. Across different models, identical prompts were used for the same task. Neither type of prompt explicitly included restrictive instructions such as “do not introduce additional knowledge.” This design was motivated by two considerations. First, prior research has shown that LLMs tend to rely heavily on contextual knowledge during generation, and that prompting models to use only the provided context can be effective [27]. Accordingly, we imposed no such restriction in the rewriting prompts intended to approximate application scenarios, while we did include a constraint in the corrective prompts requiring the model to base its output on the source text. Second, in real-world use, ordinary users rarely employ such strict negatively worded constraints. It should be noted that the absence of an explicit prohibition on external knowledge may have somewhat increased the frequency of “addition”-type distortions in our study. Even so, omission-type distortions remained predominant, indicating that this trend is relatively robust. However, compared with prompts commonly used in practice, our prompts were more structured and more explicitly task-oriented, and thus remain somewhat distant from real-world usage scenarios. Future research should systematically conduct prompt sensitivity analyses, with particular attention to two directions. First, it would be valuable to test whether our findings remain robust in more open-ended and context-rich real-world settings. Second, systematic comparisons should be made between different prompt constraint strategies—especially between prompts that simply instruct the model to rely on the source text and those that explicitly prohibit the use of external knowledge—so as to more clearly delineate the actual role of prompt constraints in shaping distortion patterns.

Additionally, this study’s experiments were based on model versions accessible between March and May 2025. As LLMs continue to evolve, their generative capabilities and behavioral patterns may change, rendering the findings of this study time-dependent to some extent. Overall, these limitations do not diminish the core findings of this study but suggest that the current results should be viewed as preliminary structural observations of LLM behavior in patient-friendly rewriting tasks. Future research requires validation of relevant conclusions through larger-scale data, more rigorous evaluation frameworks, and experimental designs that more closely approximate real-world application scenarios, thereby developing a more comprehensive understanding of the potential risks in patient-friendly rewriting by LLMs.

## 4. Methods

### 4.1. Text rewriting

#### 4.1.1. Dataset.

In this study, 100 article abstracts were manually and randomly selected from the Chemical–Disease Relations (CDR) dataset. The CDR dataset is a widely used biomedical literature corpus consisting of PubMed abstracts that focus on chemical–disease relationships. It contains 1,500 manually annotated articles in total [25]. During sampling, care was taken to avoid overrepresentation of any single disease category, in order to ensure a relatively balanced coverage of disease types. The distribution of disease categories among the 100 sampled abstracts used in this study is provided in Table 1.

**Table 1:** Distribution of disease types in our dataset.

Disease Category	Number of Abstracts
Cardiovascular diseases	20
Neurological diseases	15
Metabolic diseases	18
Tumors	12
Respiratory diseases	10
Immune system diseases	8
Other disease types	17
Total	100

### 4.2. Rewriting Procedure

#### 4.2.1. Rewriting Principles.

The rewriting task was divided into two parts: readability level rewriting and text length rewriting. Taking a sample text as an example, the original text was defined as being at the ‘current reading level with current length.’ Subsequently, this text was rewritten into two versions: one at middle school level and the other at postgraduate level. This resulted in three text reading levels (including the unchanged current level). For each reading level, the text length was then modified: each text was rewritten to its maximum length (expanded by 75%) and its minimum length (reduced by 75%), ultimately producing three text length variations (including the original length at each reading level). Fig 10 illustrates the detailed rewriting process.

**Fig 10:**
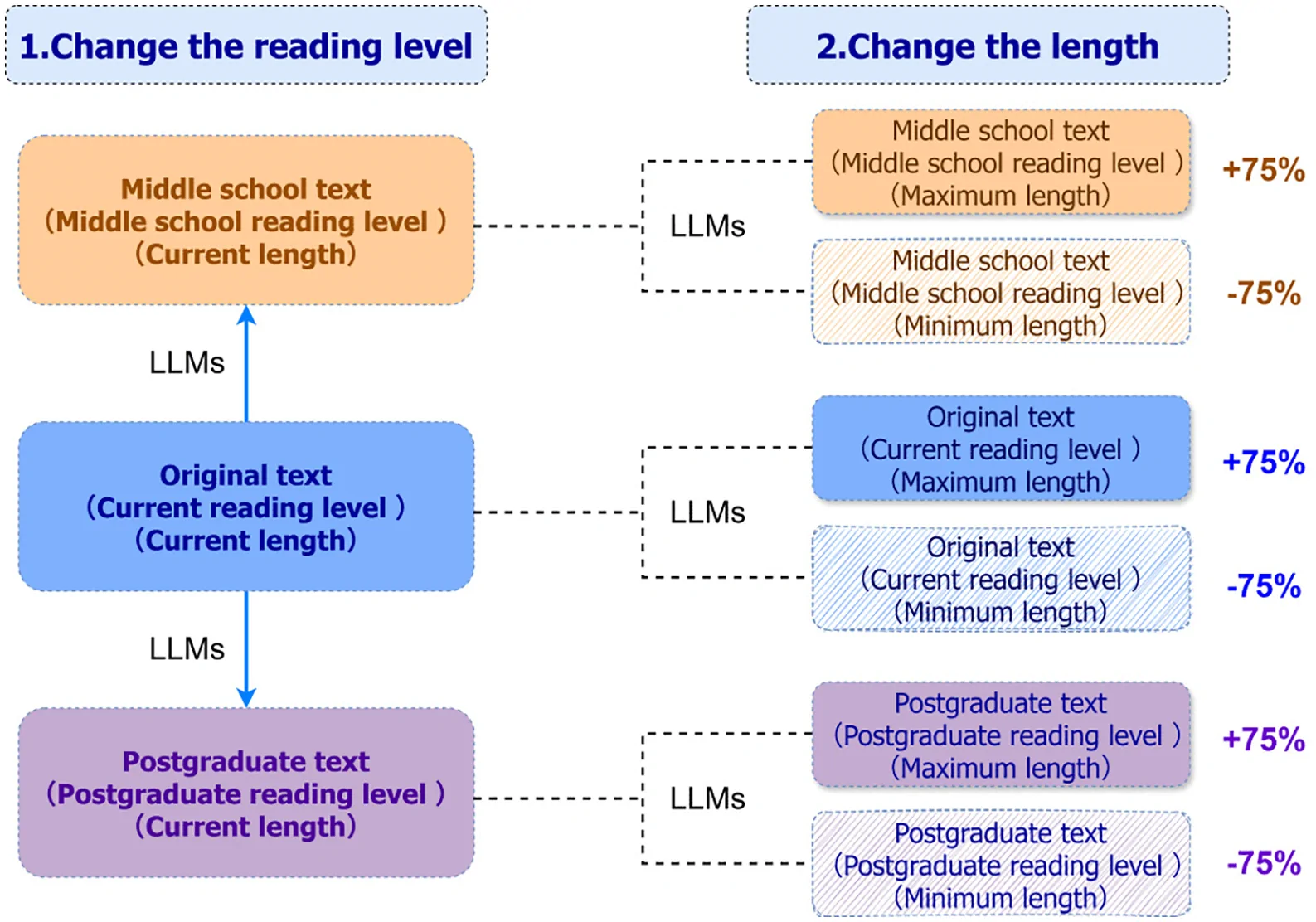
Flowchart of the rewriting process.

Two target reading levels were defined as clearly distinguishable and well-defined text-generation conditions, designed to reflect scenarios in which patients use LLMs to explain medical literature. The middle-school level produced simpler vocabulary, shorter sentences, and direct language for non-medical readers. The postgraduate level used prompts requesting advanced interpretation, generating text with specialized terminology and complex syntax for readers able to process technical language without formal medical training. These levels guided LLM text complexity and were not intended as standardized health literacy measures or to correspond to frameworks such as HLS-EU, REALM, or TOFHLA.

These two reading levels were selected for two main reasons. First, the middle school level approximates the practical reading capacity of the majority of patients and therefore represents the primary target for patient-friendly rewriting. Second, the postgraduate level represents highly educated non-specialist readers who may encounter medical literature in real-world contexts. Comparing these two levels also allowed us to examine how knowledge distortion varies across different degrees of readability adaptation.

#### 4.2.2. Analysis of rewriting effects.

Readability was evaluated using the Flesch–Kincaid Grade Level (FKGL), a commonly used metric for estimating the U.S. school grade level required to understand a given text. FKGL was calculated using the following formula: FKGL = 0.39 × (total words / total sentences) + 11.8 × (total syllables / total words) – 15.59. Target readability ranges were defined as grades 6–12 for the middle school level and grades 17–19 for the postgraduate level.

Text length variation was calculated as the percentage change in word count between the rewritten text and the original text. If the length change deviated from the target value (±75%) by more than 15%, the text was regenerated until the predefined criterion was met.

### 4.3. Model selection

#### 4.3.1. Models used in text rewriting.

The selected closed-source models were GPT-3.5-turbo and GPT-4. The selected open-source models were DeepSeek-R1, Meta-Llama-3.1-8B-Instruct, Meta-Llama-3.1-70B-Instruct, and Meta-Llama-3.3-70B-Instruct. Details of the models are presented in Table 2.

**Table 2:** Details of the models used in this study.

Model	Provider	Version	Access Method	Access Time	Main Parameter Settings
GPT-3.5-turbo	OpenAI	gpt-3.5-turbo-0613	API	March–May, 2025	temperature=0.3, Max tokens=1500
GPT-4	OpenAI	gpt-4–0613			
DeepSeek-R1	DeepSeek	deepseek-chat			
Llama-3.1-8B	Meta	Meta-Llama-3.1-8B-Instruct			
Llama-3.1-70B	Meta	Meta-Llama-3.1-70B-Instruct			
Llama-3.3-70B	Meta	Meta-Llama-3.3-70B-Instruct			

#### 4.3.2. Model used in other processes.

Based on findings from our previous evaluation study [26], related research [28,29], and preliminary experiments, GPT-4 was selected to perform extraction, comparison, and classification.

### 4.4. Triplet extraction, comparison and classification

#### 4.4.1. Triplet definition and format.

In this study, a triplet was defined as a structured representation in the form of [disease, related-relation, factor]. The disease denotes a specific disease entity explicitly mentioned in the text. The relation was uniformly represented as “related to” to indicate an association between the disease and a corresponding factor. The factor refers to any element associated with the disease.

#### 4.4.2. Triplet extraction process.

Triplets were automatically extracted using the GPT-4 model with predefined prompts (see Supplementary Information for prompt details). The prompts incorporated the following constraints: 1) disease entities had to be explicitly mentioned in the source text; 2) during relation extraction, causal relationships were prioritized, followed by associative relationships; 3) all extracted terms were required to avoid vague or ambiguous expressions; and 4) each triplet was required to be unique within a given text.

#### 4.4.3. Semantic consistency analysis.

Semantic consistency analysis was conducted to determine whether triplets identified as added in the rewritten texts were semantically equivalent to knowledge in the original texts. The procedure consisted of three steps. First, each added triplet extracted from the rewritten text was paired with all triplets extracted from the corresponding original text. Second, GPT-4 was used to evaluate semantic equivalence between paired triplets according to three criteria: 1) whether the disease entities referred to the same disease, considering synonyms and hyponym–hypernym relationships; 2) whether the factors conveyed the same meaning despite differences in wording; and3) whether the relation types were consistent. Finally, added triplets judged to be semantically equivalent to any original triplet were reclassified as consistent triplets, thereby excluding knowledge that represented paraphrased rather than genuinely new content.

#### 4.4.4. Triplet comparison.

Triplet comparison followed two principles. For readability-level rewriting, triplets extracted from the rewritten text were compared with those from the original text. For length-modification rewriting, comparisons were performed within the same readability level (e.g., the middle-school expanded version was compared with the middle-school original version).

#### 4.4.5. Triplet categorization.

Omitted and added triplets were categorized into five classes using the GPT-4 model: 1) Category E (etiology and pathophysiology), triplets in which the factor referred to disease causes, pathogenesis, or pathological processes; 2) Category S (symptoms), triplets in which the factor referred to clinical manifestations or signs; 3) Category D (disease diagnosis), triplets in which the factor referred to diagnostic methods, diagnostic criteria, or differential diagnosis. In addition, cases in which the rewritten text introduced a new disease entity not mentioned in the original text were also assigned to this category; 4) Category T (therapeutic drugs), triplets in which the factor referred to therapeutic drugs, medication regimens, mechanisms of action, or contraindications; and 5) Category C (complications and risk factors), triplets in which the factor referred to complications, prognosis-related factors, or health behavior influences. For cases that could belong to multiple categories, classification followed the priority order: E > C > T > D > S. Remaining ambiguous cases were resolved through manual adjudication.

#### 4.4.6. Prompt engineering.

Prompts were designed using a multi-level rule–based constraint framework, with the full prompt templates provided in the S1 Text. The prompt design incorporated the following elements: 1) role definition, specifying the model’s task role; 2) output format constraints, requiring results to be returned in JSON format to avoid extraneous text generation; 3) multi-level rules establishing constraints ranging from high-level principles to detailed operational instructions; 4) examples and counter-examples, providing both correct and incorrect instances to guide the model’s behavior; and 5) consistency requirements, emphasizing terminology standardization and removal of duplicate outputs.

#### 4.4.7. Manual validation.

To ensure the reliability of the results, manual validation was conducted during the processes of extraction, comparison, and classification (corresponding to Sections 2.2–2.5). For each step, 30% of the outputs were randomly sampled and independently evaluated by two researchers to assess the accuracy of the outputs generated by the LLMs. During this process, the researchers directly assessed whether each output was correct or incorrect based on the definitions and criteria specified for each task. Across all steps, the observed error rate was below 10%. All identified errors were manually corrected, and the corrected results were incorporated into the final dataset.

## Supporting information

S1 Fig Counts of added, omitted and consistent triplets after readability rewriting without semantic consistency analysis. (TIF)

S2 Fig Counts of added, omitted and consistent triplets after text-length rewriting without semantic consistency analysis. (TIF)

S3 Fig Results of semantic consistency analysis for added triplets. Panel (a) shows the proportion of consistency in added triplets across different models at middle-school and postgraduate reading levels.
(TIF)

S4 Fig Change (%) in the proportion of semantic consistency in added triplets across different scenarios after correction, relative to the pre-correction baseline. (TIF)

S5 Fig Stability analysis of knowledge distortion patterns across increasing sample sizes. (TIF)

S1 Table Readability and Length Metrics Across Rewriting Scenarios. (DOCX)

S2 Table Counts of added, omitted, and consistent triplets before correction. (DOCX)

S3 Table Counts of added, omitted, and consistent triplets after correction. (DOCX)

S4 Table Classification statistics of added triplets generated by each model after rewriting. (DOCX)

S5 Table Classification statistics of added triplets after correction. (DOCX)

S6 Table Classification statistics of omitted triplets generated by each model after rewriting. (DOCX)

S7 Table Classification statistics of omitted triplets after correction. (DOCX)

S1 Text Prompts used in this study. (DOCX)
