# Supplementary material for: Prevalent knowledge omissions: An unseen risk of LLMs in patient-friendly adaptation of medical literature
Source: PLOS Digit Health. 2026 Oct 15;5(10):e0001739. doi: 10.1371/journal.pdig.0001739 (PMC13671536; doi:10.1371/journal.pdig.0001739)
Supplement: Supplementary file 1 [file pdig.0001739.s006.docx]

Table S1 Readability and Length Metrics Across Rewriting Scenarios

| Model | Read_Level | Metric | C | C+MAX | C+MIN | M | M+MAX | M+MIN | P | P+MAX | P+MIN |
| --- | --- | --- | --- | --- | --- | --- | --- | --- | --- | --- | --- |
| DeepSeek-R1 | FKGL | Mean | 14.6 | 19.1 | 14.8 | 10 | 9.6 | 8.5 | 19.3 | 20.9 | 18.9 |
| DeepSeek-R1 | FKGL | SD | 2.5 | 2.4 | 2.6 | 1.8 | 1.2 | 1.8 | 2.4 | 2.6 | 3 |
| GPT-3.5-turbo | FKGL | Mean | 14.6 | 15.5 | 13.3 | 10.2 | 12.3 | 9.8 | 16.5 | 17.1 | 14.5 |
| GPT-3.5-turbo | FKGL | SD | 2.5 | 2.6 | 2.7 | 2.3 | 2 | 3.1 | 2.4 | 2.4 | 3.2 |
| GPT-4 | FKGL | Mean | 14.6 | 14.3 | 13.6 | 10.1 | 12.5 | 9.9 | 16.8 | 15.4 | 15 |
| GPT-4 | FKGL | SD | 2.5 | 2 | 2.9 | 1.9 | 1.7 | 2.9 | 2.7 | 2 | 3.2 |
| Llama-3.1-70B | FKGL | Mean | 14.6 | 16.5 | 15.1 | 10.1 | 11.4 | 9.8 | 17.8 | 19 | 16.7 |
| Llama-3.1-70B | FKGL | SD | 2.5 | 2.3 | 3.9 | 1.7 | 1.9 | 2.9 | 2.8 | 2.9 | 3.7 |
| Llama-3.1-8B | FKGL | Mean | 14.6 | 15 | 14.1 | 9.6 | 9.6 | 9.1 | 16.7 | 17.2 | 16.9 |
| Llama-3.1-8B | FKGL | SD | 2.5 | 2.1 | 2.7 | 1.4 | 1.5 | 1.8 | 2.5 | 2.4 | 3 |
| Llama-3.3-70B | FKGL | Mean | 14.6 | 16.6 | 15.4 | 10.2 | 11.3 | 9.9 | 18 | 18.8 | 16.8 |
| Llama-3.3-70B | FKGL | SD | 2.5 | 2.4 | 3.9 | 1.7 | 1.8 | 3.1 | 2.7 | 2.7 | 4.1 |
| DeepSeek-R1 | WordCount | Mean | 166 | 425 | 73 | 171 | 511 | 88 | 187 | 559 | 113 |
| DeepSeek-R1 | WordCount | SD | 93 | 168 | 48 | 46 | 143 | 34 | 55 | 129 | 39 |
| GPT-3.5-turbo | WordCount | Mean | 166 | 180 | 76 | 127 | 159 | 51 | 147 | 191 | 63 |
| GPT-3.5-turbo | WordCount | SD | 93 | 100 | 57 | 64 | 100 | 43 | 73 | 101 | 42 |
| GPT-4 | WordCount | Mean | 166 | 222 | 53 | 167 | 222 | 46 | 168 | 219 | 52 |
| GPT-4 | WordCount | SD | 93 | 91 | 38 | 73 | 95 | 28 | 82 | 98 | 31 |
| Llama-3.1-70B | WordCount | Mean | 166 | 182 | 30 | 151 | 170 | 22 | 149 | 169 | 26 |
| Llama-3.1-70B | WordCount | SD | 93 | 100 | 24 | 57 | 73 | 13 | 62 | 77 | 17 |
| Llama-3.1-8B | WordCount | Mean | 166 | 186 | 71 | 132 | 144 | 54 | 129 | 157 | 56 |
| Llama-3.1-8B | WordCount | SD | 93 | 108 | 38 | 49 | 67 | 20 | 47 | 68 | 22 |
| Llama-3.3-70B | WordCount | Mean | 166 | 198 | 30 | 150 | 167 | 23 | 150 | 173 | 26 |
| Llama-3.3-70B | WordCount | SD | 93 | 154 | 23 | 56 | 73 | 13 | 61 | 79 | 17 |
