# Supplementary material for: Prevalent knowledge omissions: An unseen risk of LLMs in patient-friendly adaptation of medical literature
Source: PLOS Digit Health. 2026 Oct 15;5(10):e0001739. doi: 10.1371/journal.pdig.0001739 (PMC13671536; doi:10.1371/journal.pdig.0001739)
Supplement: Supplementary file 2 [file pdig.0001739.s007.docx]

Table S2 Counts of added, omitted, and consistent triplets before correction

| **Model** | **AOC** | **M** | **P** | **C+MAX** | **C+MIN** | **M+MAX** | **M+MIN** | **P+MAX** | **P+MIN** |
| --- | --- | --- | --- | --- | --- | --- | --- | --- | --- |
| **DeepSeek** | **added** | 69 | 68 | 217 | 51 | 113 | 48 | 368 | 70 |
|  | **omitted** | 335 | 325 | 320 | 312 | 261 | 276 | 352 | 343 |
|  | **consistent** | 242 | 319 | 391 | 244 | 295 | 235 | 381 | 298 |
| **GPT-4** | **added** | 68 | 50 | 80 | 54 | 45 | 43 | 90 | 49 |
|  | **omitted** | 392 | 347 | 317 | 364 | 301 | 331 | 324 | 381 |
|  | **consistent** | 322 | 388 | 363 | 243 | 377 | 209 | 370 | 267 |
| **GPT-3.5-turbo** | **added** | 76 | 47 | 54 | 33 | 46 | 36 | 40 | 35 |
|  | **omitted** | 409 | 349 | 322 | 371 | 279 | 301 | 311 | 350 |
|  | **consistent** | 292 | 387 | 410 | 304 | 322 | 223 | 434 | 303 |
| **Llama3.1-8b** | **added** | 61 | 61 | 85 | 33 | 61 | 39 | 60 | 46 |
|  | **omitted** | 403 | 330 | 256 | 334 | 199 | 251 | 279 | 372 |
|  | **consistent** | 80 | 153 | 239 | 121 | 185 | 111 | 229 | 122 |
| **Llama3.1-70b** | **added** | 63 | 47 | 30 | 57 | 71 | 39 | 65 | 29 |
|  | **omitted** | 396 | 318 | 303 | 382 | 233 | 299 | 289 | 372 |
|  | **consistent** | 286 | 378 | 440 | 182 | 305 | 160 | 421 | 200 |
| **Llama3.3-70b** | **added** | 64 | 45 | 23 | 45 | 63 | 38 | 62 | 29 |
|  | **omitted** | 389 | 343 | 314 | 378 | 249 | 319 | 278 | 363 |
|  | **consistent** | 292 | 364 | 416 | 199 | 321 | 167 | 415 | 196 |
