# Supplementary material for: Prevalent knowledge omissions: An unseen risk of LLMs in patient-friendly adaptation of medical literature
Source: PLOS Digit Health. 2026 Oct 15;5(10):e0001739. doi: 10.1371/journal.pdig.0001739 (PMC13671536; doi:10.1371/journal.pdig.0001739)
Supplement: Supplementary file 3 [file pdig.0001739.s008.docx]

Table S3 Counts of added, omitted, and consistent triplets after correction

| **Model** | **AOC** | **M** | **P** | **C+MAX** | **C+MIN** | **M+MAX** | **M+MIN** | **P+MAX** | **P+MIN** |
| --- | --- | --- | --- | --- | --- | --- | --- | --- | --- |
| **DeepSeek** | **added** | 75 | 58 | 150 | 63 | 95 | 69 | 176 | 73 |
|  | **omitted** | 322 | 309 | 314 | 318 | 295 | 315 | 354 | 354 |
|  | **consistent** | 302 | 345 | 361 | 274 | 334 | 257 | 336 | 291 |
| **GPT-4** | **added** | 70 | 67 | 64 | 49 | 93 | 56 | 78 | 45 |
|  | **omitted** | 320 | 286 | 267 | 311 | 314 | 309 | 304 | 330 |
|  | **consistent** | 285 | 308 | 370 | 226 | 349 | 203 | 360 | 222 |
| **GPT-3.5-turbo** | **added** | 57 | 56 | 55 | 38 | 61 | 53 | 61 | 47 |
|  | **omitted** | 316 | 271 | 257 | 283 | 278 | 272 | 295 | 291 |
|  | **consistent** | 249 | 299 | 329 | 269 | 284 | 244 | 338 | 259 |
| **Llama3.1-8b** | **added** | 72 | 61 | 82 | 48 | 90 | 50 | 98 | 51 |
|  | **omitted** | 359 | 296 | 295 | 324 | 292 | 294 | 315 | 332 |
|  | **consistent** | 244 | 319 | 337 | 237 | 321 | 232 | 335 | 241 |
| **Llama3.1-70b** | **added** | 74 | 54 | 46 | 54 | 76 | 42 | 55 | 42 |
|  | **omitted** | 336 | 283 | 264 | 310 | 284 | 280 | 276 | 305 |
|  | **consistent** | 244 | 313 | 322 | 243 | 333 | 221 | 349 | 245 |
| **Llama3.3-70b** | **added** | 57 | 46 | 51 | 51 | 67 | 44 | 55 | 40 |
|  | **omitted** | 320 | 287 | 255 | 301 | 257 | 261 | 280 | 302 |
|  | **consistent** | 234 | 314 | 342 | 245 | 310 | 235 | 317 | 243 |
