# Supplementary material for: Prevalent knowledge omissions: An unseen risk of LLMs in patient-friendly adaptation of medical literature
Source: PLOS Digit Health. 2026 Oct 15;5(10):e0001739. doi: 10.1371/journal.pdig.0001739 (PMC13671536; doi:10.1371/journal.pdig.0001739)
Supplement: Supplementary file 4 [file pdig.0001739.s009.docx]

Table S4 Classification statistics of added triplets generated by each model after rewriting

| **Model** | **Category** | **M** | **P** | **C+MAX** | **C+MIN** | **M+MAX** | **M+MIN** | **P+MAX** | **P+MIN** |
| --- | --- | --- | --- | --- | --- | --- | --- | --- | --- |
| **DeepSeek** | **E** | 32 | 31 | 104 | 16 | 43 | 20 | 207 | 34 |
|  | **S** | 5 | 4 | 13 | 3 | 15 | 3 | 15 | 3 |
|  | **D** | 8 | 9 | 37 | 6 | 11 | 4 | 53 | 10 |
|  | **T** | 9 | 8 | 19 | 6 | 14 | 4 | 31 | 4 |
|  | **C** | 12 | 12 | 37 | 17 | 25 | 15 | 56 | 17 |
| **GPT-3.5**  **turbo** | **E** | 34 | 20 | 22 | 12 | 23 | 14 | 23 | 19 |
|  | **S** | 6 | 0 | 4 | 2 | 5 | 2 | 0 | 2 |
|  | **D** | 10 | 11 | 7 | 6 | 6 | 6 | 7 | 5 |
|  | **T** | 12 | 5 | 5 | 4 | 3 | 5 | 3 | 2 |
|  | **C** | 9 | 9 | 14 | 5 | 8 | 5 | 6 | 5 |
| **GPT-4** | **E** | 30 | 16 | 37 | 21 | 36 | 16 | 27 | 21 |
|  | **S** | 4 | 5 | 5 | 2 | 7 | 2 | 0 | 1 |
|  | **D** | 10 | 9 | 11 | 9 | 14 | 11 | 9 | 2 |
|  | **T** | 3 | 7 | 9 | 3 | 5 | 5 | 6 | 7 |
|  | **C** | 17 | 10 | 15 | 18 | 12 | 8 | 14 | 13 |
| **Llama3.1-8b** | **E** | 26 | 29 | 37 | 14 | 21 | 12 | 24 | 15 |
|  | **S** | 4 | 8 | 9 | 1 | 6 | 9 | 3 | 2 |
|  | **D** | 7 | 8 | 13 | 7 | 9 | 3 | 11 | 5 |
|  | **T** | 10 | 4 | 3 | 2 | 3 | 2 | 4 | 7 |
|  | **C** | 11 | 10 | 18 | 8 | 17 | 8 | 17 | 12 |
| **Llama3.1-70b** | **E** | 24 | 20 | 20 | 11 | 23 | 14 | 26 | 12 |
|  | **S** | 4 | 4 | 9 | 2 | 6 | 2 | 1 | 2 |
|  | **D** | 7 | 5 | 6 | 6 | 11 | 3 | 12 | 1 |
|  | **T** | 11 | 6 | 4 | 3 | 10 | 3 | 6 | 5 |
|  | **C** | 14 | 9 | 9 | 3 | 18 | 11 | 17 | 6 |
| **Llama3.3-70b** | **E** | 25 | 22 | 22 | 8 | 26 | 17 | 33 | 11 |
|  | **S** | 6 | 1 | 2 | 1 | 10 | 2 | 0 | 1 |
|  | **D** | 6 | 8 | 9 | 3 | 9 | 2 | 10 | 2 |
|  | **T** | 7 | 5 | 3 | 3 | 4 | 3 | 10 | 5 |
|  | **C** | 13 | 8 | 9 | 6 | 10 | 10 | 9 | 6 |
