# Supplementary material for: Prevalent knowledge omissions: An unseen risk of LLMs in patient-friendly adaptation of medical literature
Source: PLOS Digit Health. 2026 Oct 15;5(10):e0001739. doi: 10.1371/journal.pdig.0001739 (PMC13671536; doi:10.1371/journal.pdig.0001739)
Supplement: Supplementary file 5 [file pdig.0001739.s010.docx]

Table S5 Classification statistics of added triplets after correction

| **Model** | **Category** | **M** | **P** | **C+MAX** | **C+MIN** | **M+MAX** | **M+MIN** | **P+MAX** | **P+MIN** |
| --- | --- | --- | --- | --- | --- | --- | --- | --- | --- |
| **DeepSeek** | **E** | 29 | 26 | 50 | 26 | 37 | 31 | 91 | 27 |
|  | **S** | 8 | 2 | 7 | 4 | 7 | 3 | 6 | 4 |
|  | **D** | 7 | 8 | 24 | 7 | 11 | 7 | 20 | 6 |
|  | **T** | 6 | 7 | 19 | 5 | 8 | 6 | 11 | 9 |
|  | **C** | 21 | 15 | 45 | 18 | 28 | 18 | 41 | 24 |
| **GPT-3.5**  **turbo** | **E** | 25 | 19 | 21 | 16 | 32 | 23 | 27 | 15 |
|  | **S** | 3 | 1 | 2 | 0 | 2 | 4 | 0 | 3 |
|  | **D** | 4 | 10 | 6 | 5 | 6 | 6 | 5 | 5 |
|  | **T** | 6 | 7 | 10 | 2 | 2 | 4 | 10 | 5 |
|  | **C** | 16 | 19 | 14 | 14 | 15 | 7 | 15 | 16 |
| **GPT-4** | **E** | 36 | 34 | 25 | 18 | 33 | 24 | 36 | 15 |
|  | **S** | 6 | 2 | 3 | 3 | 6 | 3 | 1 | 2 |
|  | **D** | 2 | 5 | 14 | 5 | 15 | 6 | 8 | 7 |
|  | **T** | 5 | 6 | 8 | 6 | 8 | 7 | 13 | 7 |
|  | **C** | 14 | 18 | 11 | 15 | 26 | 13 | 17 | 10 |
| **Llama3.1-8b** | **E** | 22 | 25 | 42 | 20 | 45 | 22 | 40 | 22 |
|  | **S** | 8 | 4 | 4 | 3 | 5 | 5 | 2 | 3 |
|  | **D** | 8 | 9 | 7 | 3 | 9 | 2 | 14 | 3 |
|  | **T** | 9 | 5 | 9 | 8 | 11 | 9 | 7 | 6 |
|  | **C** | 25 | 17 | 19 | 12 | 17 | 10 | 13 | 16 |
| **Llama3.1-70b** | **E** | 32 | 17 | 19 | 16 | 42 | 16 | 28 | 20 |
|  | **S** | 9 | 4 | 6 | 3 | 5 | 3 | 2 | 4 |
|  | **D** | 6 | 2 | 4 | 5 | 6 | 3 | 4 | 2 |
|  | **T** | 5 | 8 | 3 | 5 | 6 | 3 | 6 | 6 |
|  | **C** | 17 | 20 | 13 | 22 | 14 | 12 | 8 | 9 |
| **Llama3.3-70b** | **E** | 25 | 24 | 21 | 23 | 25 | 21 | 30 | 15 |
|  | **S** | 3 | 1 | 2 | 1 | 1 | 0 | 1 | 1 |
|  | **D** | 6 | 2 | 8 | 7 | 6 | 3 | 7 | 5 |
|  | **T** | 3 | 8 | 7 | 8 | 8 | 6 | 3 | 5 |
|  | **C** | 17 | 11 | 12 | 8 | 24 | 10 | 10 | 10 |
