# Supplementary material for: Prevalent knowledge omissions: An unseen risk of LLMs in patient-friendly adaptation of medical literature
Source: PLOS Digit Health. 2026 Oct 15;5(10):e0001739. doi: 10.1371/journal.pdig.0001739 (PMC13671536; doi:10.1371/journal.pdig.0001739)
Supplement: Supplementary file 6 [file pdig.0001739.s011.docx]

Table S6 Classification statistics of omitted triplets generated by each model after rewriting

| **Model** | **Category** | **M** | **P** | **C+MAX** | **C+MIN** | **M+MAX** | **M+MIN** | **P+MAX** | **P+MIN** |
| --- | --- | --- | --- | --- | --- | --- | --- | --- | --- |
| **DeepSeek** | **E** | 149 | 151 | 134 | 148 | 125 | 107 | 168 | 156 |
|  | **S** | 19 | 13 | 16 | 15 | 16 | 19 | 13 | 12 |
|  | **D** | 16 | 15 | 19 | 9 | 9 | 13 | 22 | 26 |
|  | **T** | 59 | 54 | 58 | 51 | 43 | 48 | 52 | 48 |
|  | **C** | 81 | 77 | 83 | 78 | 61 | 78 | 83 | 84 |
| **GPT-3.5**  **turbo** | **E** | 176 | 163 | 130 | 171 | 123 | 141 | 142 | 166 |
|  | **S** | 18 | 18 | 12 | 19 | 13 | 17 | 8 | 10 |
|  | **D** | 33 | 21 | 23 | 20 | 16 | 20 | 21 | 22 |
|  | **T** | 68 | 57 | 60 | 62 | 39 | 45 | 56 | 65 |
|  | **C** | 93 | 70 | 78 | 78 | 74 | 65 | 69 | 73 |
| **GPT-4** | **E** | 191 | 162 | 147 | 177 | 123 | 145 | 140 | 163 |
|  | **S** | 12 | 10 | 9 | 12 | 16 | 17 | 11 | 22 |
|  | **D** | 21 | 18 | 14 | 11 | 23 | 24 | 13 | 23 |
|  | **T** | 63 | 66 | 64 | 67 | 58 | 60 | 75 | 79 |
|  | **C** | 90 | 77 | 72 | 83 | 71 | 70 | 72 | 84 |
| **Llama3.1-8b** | **E** | 181 | 147 | 111 | 146 | 82 | 110 | 136 | 190 |
|  | **S** | 16 | 14 | 14 | 13 | 12 | 12 | 12 | 20 |
|  | **D** | 27 | 21 | 14 | 21 | 10 | 11 | 13 | 16 |
|  | **T** | 63 | 63 | 43 | 55 | 46 | 47 | 59 | 58 |
|  | **C** | 98 | 74 | 68 | 89 | 42 | 60 | 52 | 71 |
| **Llama3.1-70b** | **E** | 190 | 153 | 144 | 174 | 104 | 122 | 127 | 159 |
|  | **S** | 19 | 5 | 4 | 15 | 15 | 18 | 12 | 18 |
|  | **D** | 15 | 12 | 21 | 25 | 6 | 14 | 15 | 24 |
|  | **T** | 64 | 49 | 44 | 63 | 40 | 51 | 53 | 78 |
|  | **C** | 88 | 81 | 72 | 85 | 57 | 71 | 67 | 76 |
| **Llama3.3-70b** | **E** | 186 | 158 | 153 | 170 | 102 | 132 | 135 | 181 |
|  | **S** | 15 | 15 | 16 | 16 | 21 | 30 | 8 | 6 |
|  | **D** | 20 | 25 | 16 | 24 | 7 | 9 | 15 | 23 |
|  | **T** | 59 | 57 | 46 | 54 | 46 | 52 | 46 | 59 |
|  | **C** | 94 | 75 | 68 | 97 | 62 | 84 | 64 | 77 |
