# Supplementary material for: Prevalent knowledge omissions: An unseen risk of LLMs in patient-friendly adaptation of medical literature
Source: PLOS Digit Health. 2026 Oct 15;5(10):e0001739. doi: 10.1371/journal.pdig.0001739 (PMC13671536; doi:10.1371/journal.pdig.0001739)
Supplement: Supplementary file 7 [file pdig.0001739.s012.docx]

Table S7 Classification statistics of omitted triplets after correction

| **Model** | **Category** | **M** | **P** | **C+MAX** | **C+MIN** | **M+MAX** | **M+MIN** | **P+MAX** | **P+MIN** |
| --- | --- | --- | --- | --- | --- | --- | --- | --- | --- |
| **DeepSeek** | **E** | 158 | 165 | 156 | 162 | 156 | 154 | 167 | 181 |
|  | **S** | 9 | 7 | 8 | 7 | 18 | 23 | 19 | 15 |
|  | **D** | 12 | 18 | 15 | 18 | 10 | 18 | 20 | 19 |
|  | **T** | 51 | 46 | 51 | 44 | 41 | 45 | 52 | 55 |
|  | **C** | 89 | 79 | 88 | 81 | 76 | 91 | 96 | 84 |
| **GPT-3.5**  **turbo** | **E** | 141 | 120 | 113 | 133 | 124 | 127 | 116 | 126 |
|  | **S** | 10 | 5 | 4 | 8 | 16 | 15 | 14 | 11 |
|  | **D** | 12 | 9 | 10 | 6 | 13 | 13 | 18 | 18 |
|  | **T** | 52 | 43 | 52 | 40 | 42 | 42 | 62 | 48 |
|  | **C** | 85 | 78 | 62 | 79 | 70 | 64 | 70 | 81 |
| **GPT-4** | **E** | 148 | 119 | 124 | 149 | 153 | 153 | 126 | 149 |
|  | **S** | 16 | 16 | 15 | 16 | 16 | 18 | 14 | 7 |
|  | **D** | 24 | 16 | 13 | 14 | 15 | 11 | 21 | 22 |
|  | **T** | 51 | 48 | 47 | 49 | 44 | 41 | 46 | 54 |
|  | **C** | 66 | 71 | 61 | 70 | 78 | 76 | 83 | 80 |
| **Llama3.1-8b** | **E** | 170 | 139 | 141 | 145 | 121 | 134 | 131 | 149 |
|  | **S** | 19 | 13 | 15 | 20 | 18 | 19 | 7 | 9 |
|  | **D** | 25 | 21 | 22 | 22 | 21 | 19 | 24 | 28 |
|  | **T** | 50 | 54 | 43 | 50 | 46 | 51 | 47 | 49 |
|  | **C** | 80 | 61 | 61 | 73 | 73 | 53 | 92 | 84 |
| **Llama3.1-70b** | **E** | 164 | 130 | 106 | 146 | 134 | 137 | 120 | 131 |
|  | **S** | 13 | 15 | 12 | 17 | 16 | 12 | 13 | 17 |
|  | **D** | 21 | 14 | 20 | 18 | 25 | 19 | 20 | 18 |
|  | **T** | 65 | 54 | 45 | 51 | 37 | 37 | 37 | 55 |
|  | **C** | 59 | 61 | 65 | 61 | 62 | 57 | 71 | 77 |
| **Llama3.3-70b** | **E** | 142 | 135 | 114 | 139 | 128 | 124 | 118 | 138 |
|  | **S** | 9 | 8 | 7 | 13 | 13 | 9 | 13 | 16 |
|  | **D** | 19 | 17 | 14 | 13 | 19 | 20 | 18 | 12 |
|  | **T** | 61 | 51 | 47 | 56 | 34 | 30 | 53 | 58 |
|  | **C** | 78 | 64 | 59 | 64 | 54 | 65 | 70 | 67 |
