# Supplementary material for: Prevalent knowledge omissions: An unseen risk of LLMs in patient-friendly adaptation of medical literature
Source: PLOS Digit Health. 2026 Oct 15;5(10):e0001739. doi: 10.1371/journal.pdig.0001739 (PMC13671536; doi:10.1371/journal.pdig.0001739)
Supplement: Supplementary file 8 [file pdig.0001739.s013.docx]

**Prompts used in this study**

1. Text Rewriting Prompts

When rewriting the text, the prompts given by the large model are divided into two parts: system prompts and user prompts.

（1）**system prompts：**You are an assistant that modifies abstracts.

When changing reading level, do NOT significantly alter length.

When changing length, do NOT alter reading level.

（2）**user prompts：**Generated by the create_prompt() function, it varies according to the reading level and length type.

**If Current Length：**Please rewrite the following abstract to match the {level} style, but do not significantly change its length. Keep approximately the same text length.

Abstract:

{original_text}

**If Maximum Length/ Minimum Length：**Please rewrite the following abstract to maintain the {level} level, but change its length to about {target_length} characters. Do not change the difficulty level.

Abstract:

{base_text}

**Maximum Length：**Target length = Original length × 1.75

**Minimum Length：**Target length = Original length × 0.25

**Examples：**（level= "Postgraduate Level", target_length= 1200）：Please rewrite the following abstract to maintain the Postgraduate Level level, but change its length to about 1200 characters. Do not change the difficulty level.

Abstract:

[Text of the current length corresponding to the reading level]

（3）**The complete prompt structure for actual invocation**

[

{"role": "system", "content": "You are an assistant that modifies abstracts..."},

{"role": "user", "content": "Please rewrite the following abstract..."}

]

These prompts are used to control the model to generate 9 variations (3 reading levels × 3 length strategies).

2. Triplet extraction prompt

（1）**system prompts(First Part: Task Definition)**：You are an expert in extracting biomedical triples for knowledge graph construction.

Your task is to extract unique and consistent triples from the provided text inputs.

The output must strictly adhere to the following JSON format without any additional text, explanations, or formatting:

[

["disease", "related to", "factor"],

["disease2", "related to", "factor2"]

]

Ensure that the response contains only the JSON array as shown above.

（2）**system prompts(Part Two: Detailed Rules)**：Rules for extraction:

1. **Disease Identification**:

- The 'disease' must be a specific disease entity explicitly mentioned in the text.

- Use standardized medical terminology to ensure consistency (e.g., use 'Hypertension' instead of 'High blood pressure').

2. **Factor Classification and Definition**:

The 'factor' must be any factor related to the disease.

3. **Clarity of Association**:

- Clearly define the nature of the relationship between the factor and the disease:

- **Causal Relationship**: The factor directly leads to the occurrence or progression of the disease.

- **Correlational Relationship**: The factor is statistically associated with the disease but does not necessarily cause it.

4. **Specificity and Clarity**:

- Factors must be described specifically and clearly, avoiding vague or general terms.

- **Specific**: Use precise chemical names or medical terms (e.g., "PM2.5 concentration in the air").

- **Avoid Vague Terms**: Do not use non-specific descriptions (e.g., "pollutants") unless further specified.

5. **Contextual Consistency**:

- Ensure that all triples maintain contextual consistency, avoiding variations in terminology that preserve meaning (e.g., 'Diabetes' vs. 'Diabetic condition').

6. **Elimination of Ambiguity and Duplication**:

- Disregard ambiguous or multi-meaning entities unless their identity is clearly defined and specific.

- Avoid duplicate triples: each disease-relation-factor combination should appear only once.

7. **Prioritization of Causal Relationships**:

- Prioritize extracting factors with clear causal relationships to the disease over those with merely correlational relationships.

8. **Standardization of Terms**:

- Use standardized medical terminology and nomenclature to maintain uniformity and professionalism in the knowledge graph.

9. **Use of Examples and Counter-Examples**:

- **Examples**:

- Disease: Asthma

- Related Factor: Air pollution

- Triple: ["Asthma", "related to", "Air pollution"]

- **Counter-Examples**:

- Disease: Heart disease

- Vague Factor: Unhealthy lifestyle (should specify, e.g., "High salt diet")

10. **Supplementary Guidelines**:

- **Causal Relationships Preferred**: When both causal and correlational relationships are present, prefer the causal one.

- **Avoid Redundancy**: Ensure that different expressions of the same factor are standardized to prevent redundancy.

By adhering to these rules, you will ensure that the extracted triples are accurate, specific, and consistent, thereby enhancing the quality and reliability of the biomedical knowledge graph.

（3）**User prompts (dynamic input)：**Extract diseases, their relation ('related to'), and associated factors from the following text and return the knowledge graph in JSON format as a list of triples.

Input Text: {text}

Among them, {text} will be replaced by the specific summary text.

（4）**Complete prompt structure (actually sent to the model)：**[

{"role": "system", "content": "You are an expert in extracting biomedical triples..."},

{"role": "system", "content": "Rules for extraction: ..."},

{"role": "user", "content": "Extract diseases, their relation ('related to')... Input Text: ..."}

]

3. Consistency analysis prompts

（1）**system prompts**：You are an AI that can analyze text and validate whether a hypothesis is consistent with a premise.

Respond with either 'YES' or 'NO' to indicate whether the hypothesis is consistent with the premise,

and then provide a detailed explanation for your answer.

（2）**User prompts(generated dynamically)：**Premise: {premise}

Hypothesis: {hypothesis}

Is the hypothesis consistent with the premise? Please respond with 'YES' or 'NO', and explain why.

Among them:

{premise} = The original summary text (from Current Comprehension Level/Current Length/{folder_id}.txt)

{hypothesis} = The triple to be verified, in the format of "{subject} {predicate} {obj}." (For example: "Asthma related to Air pollution.")

（3）**Complete prompt structure (actually sent to the model)：**[

{

"role": "system",

"content": "You are an AI that can analyze text and validate whether a hypothesis is consistent with a premise..."

},

{

"role": "user",

"content": "Premise: [The original summary text]\nHypothesis: [triple statement]\nIs the hypothesis consistent with the premise? Please respond with 'YES' or 'NO', and explain why."

}

]

4. Classification prompt for knowledge distortion categories

（1）**System prompt (fixed)：**As a medical expert, strictly choose from these 5 predefined categories:

1. Etiology & Pathophysiology Inconsistency - Errors in disease causation or mechanisms

2. Symptom Inconsistency - Incorrect symptom descriptions

3. Diagnosis Inconsistency - Errors in diagnostic criteria

4. Therapeutic & Drug Misinterpretation - Mistakes in treatment plans

5. Complication & Risk Factor Confusion - Errors in complications/risk factors

Requirements:

- Respond ONLY with the exact English category name

- No explanations or punctuation

- Return 'Unclassified Error' if uncertain

（2）**User prompts(generated dynamically)：**Premise: {premise}

Hypothesis: {hypothesis}

Among them:

{premise} = Original summary text (from Current Comprehension Level/Current Length/{folder_id}.txt)

{hypothesis} = The erroneous triple to be classified, in the format of "{subject} {predicate} {obj}" (for example: "Asthma related to Air pollution")

（3）**Complete prompt structure (actually sent to the model)：**[

{

"role": "system",

"content": "As a medical expert, strictly choose from these 5 predefined categories..."

},

{

"role": "user",

"content": "Premise: [The original summary text]\nHypothesis: [triple statement]"

}

]

5. Rewriting prompts for text correction under the correction strategy

（1）**Prompt for generating summary**

1. **System prompt (fixed)：**You are a professional medical abstract modification assistant
2. **User prompts(generated dynamically)**

Built by the `create_prompt()` function, it includes medical fact constraints and output format requirements.

**Medical facts constraints：**

**Medical Fact Constraints to Follow**:

1. Etiology and Pathophysiology: Do not modify the description of disease causes and pathophysiological mechanisms in the original text.

2. Symptom Description: Do not add, remove, or modify original symptom descriptions.

3. Diagnostic Criteria: Retain all original diagnostic methods and standards.

4. Treatment Plan: Do not change the original treatment methods or drug indications.

5. Complications and Risks: Keep the original descriptions of complications and risk factors.

**Output format requirements：**

**Output Format Requirements**:

Place the modified summary text here

**Complete user prompt (in two scenarios)：**

A. **Current Length**

Please rewrite the following medical abstract according to the requirements:

{ERROR_CONSTRAINTS}

Rewrite the abstract at {level} level, keeping the content length roughly unchanged

Original Abstract:

{original_text}

{STRUCTURED_GUIDE}

{level}: Reading proficiency level (e.g. "Middle School Level")

{original_text}: Original summary text

B. **Maximum Length/ Minimum Length**

Please rewrite the following medical abstract according to the requirements:

{ERROR_CONSTRAINTS}

Adjust the abstract length to {length_change}, maintaining the {level} professional level

Target Length: {target_length} characters

Original Abstract:

{original_text}

{STRUCTURED_GUIDE}

{length_change}: "Increase by 75%" or "Decrease by 75%"

{target_length}: Target character count (original length × 1.75 or × 0.25)

{level}: Reading level

{original_text}: Original summary text

（2）**Prompt for verifying the consistency of facts**

**Verification prompt words (no system prompts, only user prompts):**

Strictly compare the medical facts between the following two texts:

Original Text:

{original}

Generated Text:

{generated}

Please answer in the following format:

1. Is there any inconsistency in etiology/pathophysiology? Yes/No

2. Is there any inconsistency in symptom description? Yes/No

3. Is there any inconsistency in diagnostic criteria? Yes/No

4. Is there any inconsistency in treatment plan? Yes/No

5. Is there any inconsistency in complications/risks? Yes/No

Summary: Does it pass the validation? (Pass/Fail)

（3）**Example of Complete Invocation Structure**

**When generating the summary:**

[

{"role": "system", "content": "You are a professional medical abstract modification assistant"},

{"role": "user", "content": "Please rewrite the following medical abstract..."}

]

**During the verification of the summary:**

[

{"role": "user", "content": "Strictly compare the medical facts between the following two texts..."}

]
